# Diffusion and bulk flow of amino acids mediate calcium waves in plants

**DOI:** 10.1126/sciadv.abo6693

**Published:** 2022-10-21

**Authors:** Annalisa Bellandi, Diana Papp, Andrew Breakspear, Joshua Joyce, Matthew G. Johnston, Jeroen de Keijzer, Emma C. Raven, Mina Ohtsu, Thomas R. Vincent, Anthony J. Miller, Dale Sanders, Saskia A. Hogenhout, Richard J. Morris, Christine Faulkner

**Affiliations:** ^1^ Crop Genetics, John Innes Centre, Norwich Research Park, Norwich, UK.; ^2^ Biochemistry and Metabolism, John Innes Centre, Norwich Research Park, Norwich, UK.; ^3^ Computational and Systems Biology, John Innes Centre, Norwich Research Park, Norwich, UK.

## Abstract

In plants, a variety of stimuli trigger long-range calcium signals that travel rapidly along the vasculature to distal tissues via poorly understood mechanisms. Here, we use quantitative imaging and analysis to demonstrate that traveling calcium waves are mediated by diffusion and bulk flow of amino acid chemical messengers. We propose that wounding triggers release of amino acids that diffuse locally through the apoplast, activating the calcium-permeable channel GLUTAMATE RECEPTOR-LIKE 3.3 as they pass. Over long distances through the vasculature, the wound-triggered dynamics of a fluorescent tracer show that calcium waves are likely driven by bulk flow of a channel-activating chemical. We observed that multiple stimuli trigger calcium waves with similar dynamics, but calcium waves alone cannot initiate all systemic defense responses, suggesting that mobile chemical messengers are a core component of complex systemic signaling in plants.

## Teaser

Calcium waves are likely driven by chemical messengers that trigger cellular responses as they travel passively through tissues.

## INTRODUCTION

Plants exhibit systemic responses to a variety of stimuli, including pathogen and herbivore attack, osmotic stress, and wounding, yet how signals travel from the site of perception to act in distal tissues is not well understood. Wounding triggers some of the most rapid and strong of the systemic responses and is associated with both short- and long-range signaling that mediates the induction of defense responses in nonwounded cells and tissues. Calcium fluxes are transmitted across short and long distances in response to wounding and many other stresses, consistent with its function as a universal intracellular second messenger that relays information from cell to cell and tissue to tissue in all multicellular organisms. In plants, the molecular and physical processes that underpin calcium wave transmission from the site of wounding to distal tissues and the relationship of calcium waves to other traveling signals is a subject of considerable debate.

Long-distance signaling in plants is a long-observed response, and seminal work by Cunningham (*1*), Ricca (*2*), and Houwink (*3*) identified that wound-triggered signals can pass through dead tissue. These observations lead to the suggestion that systemic signals are carried by a xylem-mobile, apoplastic chemical messenger, termed the “Ricca factor.” While these studies preceded much of the knowledge of the molecules associated with signal transmission, modeling has suggested that the dynamics of systemic calcium waves could be sustained by a mobile Ricca factor (*4*). However, recent hypotheses for calcium wave transmission have emphasized a key role for hydraulic signals (*5*–*7*) and calcium-induced calcium release ion channel–mediated propagation (*8*, *9*).

Current models for systemic signaling cover a range of distinct mechanisms driven by different biophysical processes. For example, the squeeze cell hypothesis for systemic wound signaling involves a mechanism in which wound-triggered changes in xylem pressure drive ion fluxes that underlie slow wave potentials (SWPs), placing a pressure (or hydraulic) wave as the central driver of calcium and electrical signals (*5*). By contrast, calcium-induced calcium release ion channel–mediated wave propagation, proposed to explain osmotic stress triggered by calcium waves, depends on a dual calcium/reactive oxygen species (ROS) mechanism mediated by calcium channels and reduced form of nicotinamide adenine dinucleotide phosphate (NADPH) oxidases that allow the wave to actively propagate along the cellular plasma membrane (*9*). Observations that mutants with altered cytoplasmic connectivity (via altered plasmodesmal function) exhibit differences in systemic calcium signals (*6*, *10*) suggest that a soluble chemical messenger might travel cell to cell to contribute to the calcium signal, leading to integration of a symplastic intercellular signal into the dual calcium/ROS wave to propose a multicomponent transmission mechanism (*6*). These diverse models are all supported by experimental data, illustrating the complexity of long-distance signaling in plants. However, whether each model accurately represents coexisting facets of signaling is a critical question. While it is possible that a single complex mechanism for signal transmission exists, questions remain regarding whether waves triggered by different stimuli travel between cells and organs via the same mechanism, how different signals are integrated, and whether different signals carry the same information.

Investigations into rapid systemic responses use a range of experimental systems, measuring different parameters and responses, adding further complexities to the challenge of consolidating our understanding of systemic signaling in plants. Here, we have established quantitative, live imaging methods with which we can measure the dynamics of local, vascular, and distal calcium waves with high resolution. We have used these methods to characterize calcium wave dynamics in response to a range of stimuli and tested whether they are consistent with existing models of calcium wave transmission. Furthermore, we have used modeling approaches to identify the mechanisms that drive wave transmission, leading us to the conclusion that the century-old model of a xylem-mobile Ricca factor applies to this response and that transmission of calcium waves can be explained by apoplastic diffusion and bulk flow of amino acids that activate glutamate receptor-like (GLR) proteins as they pass through tissues.

## RESULTS

### Calcium waves triggered by different stimuli have similar dynamics

**Fig. 1.**
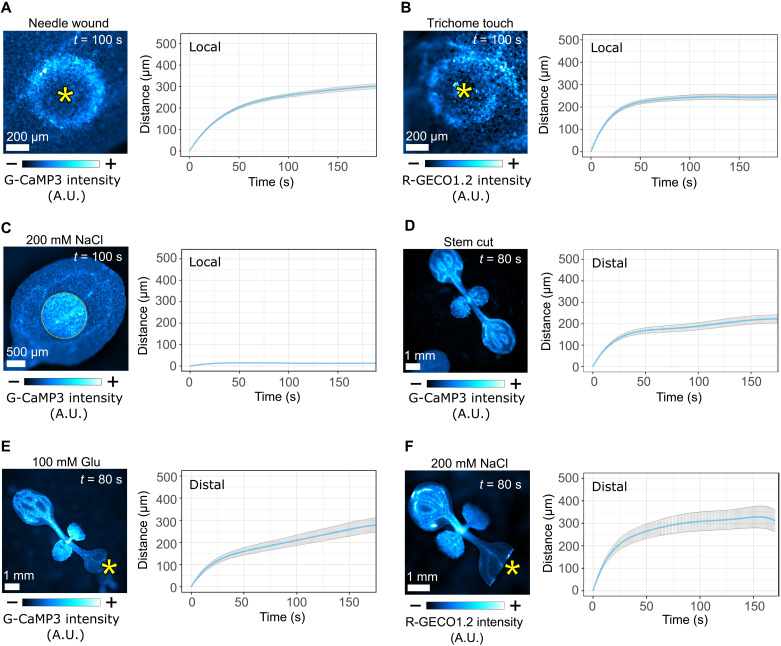
Different stimuli trigger local and distal calcium waves with similar dynamics. (**A**) Wound-triggered (*n* = 11) and (**B**) touch-triggered (*n* = 13) local calcium waves, with mean distance (±SE) over time plots for the wave peak progression. Asterisks mark the wound site or trichome base. (**C**) Application of a 200 mM NaCl droplet on the surface of a cotyledon and quantification of the calcium response (*n* = 6) with mean distance (±SE) over time plots for the wave peak progression. Distal calcium waves were triggered by (**D**) stem cutting (*n* = 13) or by application (asterisk) of (**E**) 100 mM Glu (*n* = 10) or (**F**) 200 mM NaCl (*n* = 6) on previously cut cotyledons, with mean distance (±SE) over time plots for the wave peak progression. Calibration bars indicate fluorescence intensity from low (−) to high (+). A.U., arbitrary units.

To dissect the mechanisms that drive calcium wave transmission, we first profiled the local (within tissue) calcium wave responses to a range of known stimuli (fig. S1, A to F) using plants that express a calcium reporter. Wounding and touch both triggered a radial calcium wave with a traveling peak of fluorescence that emanated from the site of stimulus and slowed with time (Fig. 1, A and B). Analysis of the dynamics of local waves identified that they have initial speeds of 7.5 (wound) and 11.8 μm/s (touch; fig. S2, A and B), similar to previous observations of aphid-triggered local calcium waves (*11*). Application of 200 mM NaCl (hereafter salt) to the leaf surface did not produce a local calcium wave (Fig. 1C). We also examined long-distance, systemic calcium waves triggered by different stimuli and here separated the wave into two phases: the primary “vascular” wave that travels along the veins and the secondary “distal” wave that subsequently emanates from the veins in nonwounded leaves, traveling from the vein into the surrounding tissues (fig. S1, G and H). As previously observed (*7*, *10*, *12*, *13*), we found that wounding, glutamate, and salt application each triggered rapid vascular waves and slower distal waves. The distal waves, similar to the local calcium responses, each have a traveling peak that slows with time (Fig. 1, D to F, and fig. S2, D to F). While we noted that salt application triggered systemic calcium waves, the waves initiated at varying locations along the veins rather than at the site of application. However, the dynamics of the salt-triggered distal waves that emanated from the veins were similar in all occurrences (Fig. 1F). The similarity in the dynamics of local and systemic waves triggered in response to all stimuli suggests the possibility that the mechanism of wave transmission is common to different triggers. Furthermore, the observation that local and distal waves slow down point to both being driven by a passive or dissipating process such as diffusion.

### Vascular calcium waves are driven by bulk flow

**Fig. 2.**
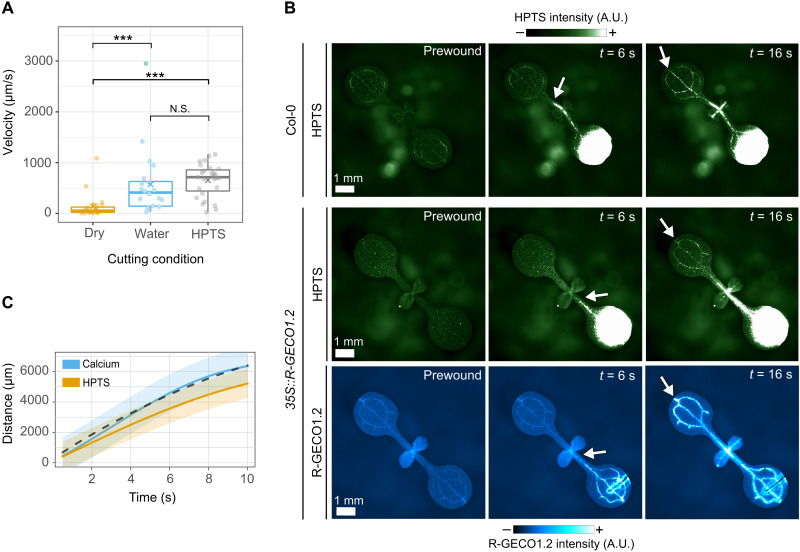
The dynamics of vascular calcium waves in response to wounding are consistent with bulk flow. (**A**) Velocity of vascular calcium waves triggered by cutting the tip of a cotyledon in a droplet water (*n* = 20), in HPTS (*n* = 27), or dry (*n* = 20). Statistical analysis by Wilcoxon rank sum test with Bonferroni correction for multiple comparisons (water versus dry, *P* = 1.6 × 10^−4^; HPTS versus dry, *P* = 2.5 × 10^−6^; water versus HPTS, *P =* 0.19; N.S., nonsignificant; ***P < 0.05). (**B**) Wound-triggered progression of HPTS and calcium along the vasculature in Col-0 plants (top) or *35S::R-GECO1.2* plants. White arrows indicate the front of the detected signals. Calibration bars indicate fluorescence intensity from low (−) to high (+). (**C**) Mean distance (±SE) traveled over time by HPTS (orange, *n* = 21) and calcium (blue, *n* = 24) computed using a GP model. The predicted distance traveled by a wave driven by bulk flow and Taylor dispersion is shown by the black dashed line.

The vascular calcium waves, which we recorded here at approximately 100 to 500 μm/s and by others in the range 60 to 1000 μm/s (*14*), are 10 to 100 times faster than the local and distal waves. To experimentally test whether these speeds are compatible with passive transmission, we compared the speed of calcium waves with the speed of travel of the fluorescent tracer 8-hydroxypyrene-1,3,6-trisulfonic acid (HPTS) in the vasculature. During optimization of the experimental system, we observed that if we placed a drop of water or HPTS on the surface of a cotyledon and cut the leaf underneath this drop, then the vascular waves traveled significantly faster and further than when the cut was made on a dry cotyledon (Fig. 2A and fig. S3A). This suggests that the vascular calcium wave speed can be regulated independently of genetically encoded factors. When the cut was made in a drop of HPTS, we observed that both HPTS and the calcium wave travel with similar dynamics in both basipetal and acropetal directions (Fig. 2B and movie S1). As HPTS travels in the vasculature by bulk flow, the similar dynamics of the HPTS front and the calcium wave suggest that the calcium wave can be explained by bulk flow.

By contrast with the HPTS front, vascular calcium waves are likely a consequence of activation of ion channels in the cells adjacent to the flow path (xylem vessels or phloem sieve tubes) and not a direct read out of bulk flow. Mathematical models predict that the distance traveled by these waves should exceed that of bulk flow because of shear-enhanced diffusion, or Taylor dispersion, of channel-activating molecules in the flow (fig. S3C) (*4*). Thus, the vascular calcium wave is driven by the combination of bulk flow and shear-enhanced diffusion and is expected to be faster than bulk flow alone. Consistent with this, quantitative analysis of the dynamics of the vascular HPTS and calcium waves identifies that the HPTS wave (visible as it travels via bulk flow) consistently lagged behind the calcium wave (21 of the 22 replicates, *P* < 2 × 10^−5^; fig. S3B). Furthermore, fitting the data for the distance traveled over time by HPTS with a Gaussian process (GP) model and adding the predicted effect of Taylor dispersion matches the experimentally measured calcium wave (Fig. 2C and fig. S3B). Therefore, the speed of vascular calcium waves is consistent with bulk flow of a channel-activating chemical messenger.

### Wound-triggered calcium waves are not mediated by a TPC1/RBOHD-mediated dual calcium/ROS wave

**Fig. 3.**
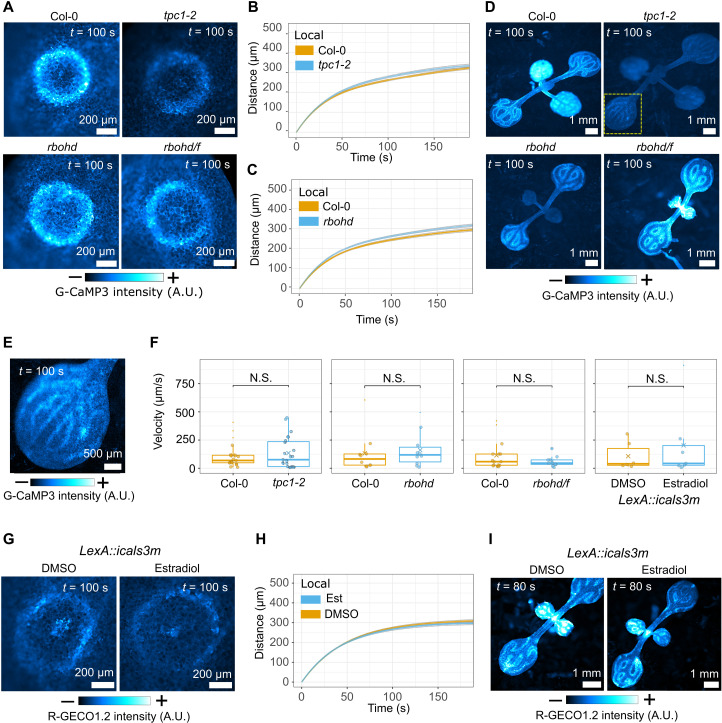
Local, distal, and vascular wound–induced calcium waves are independent from *TPC1*, *RBOHD*, *RBOHF*, and cytoplasmic connectivity. (**A**) Local wound–triggered calcium waves in tpc1, rbohd, and rbohd/f mutants with mean distance (±SE) over time plots for the wave peak progression of local waves for (**B**) tpc1-2 (*n* = 18) and (**C**) rbohd (*n* = 20) compared to Col-0 (*n* = 20 in each experiment). Data were statistically analyzed at each time point with Student’s *t* test (*P* > 0.05 for all time points). (**D**) Distal wound–triggered calcium waves in tpc1-2, rbohd, and rbohd/f mutants. (**E**) Enlarged detail of the yellow square in (D) with enhanced brightness/contrast levels. (**F**) Mean velocity of vascular calcium waves tpc1-2 (*n* = 23), rbohd (*n* = 10), and rbohd/f (*n* = 10) mutants compared to Col-0 (*n* = 19, 10, and 15, respectively) and induced (estradiol treated, *n* = 7) and uninduced [dimethyl sulfoxide (DMSO) treated, *n* = 6] LexA::icals3m. Data were analyzed by a Wilcoxon rank sum test; *P* > 0.05 for each comparison indicated by N.S. (Col-0 versus tpc1-2, *P* = 0.9; Col-0 versus rbohd, *P* = 0.6; Col-0 versus rbohd/f, *P* = 0.6; LexA::icals3m estradiol versus DMSO, *P* = 1). (**G**) Local and (**I**) distal wound–triggered calcium waves in induced and uninduced LexA::icals3m with (**H**) mean distance (±SE) over time plot for the wave peak progression of local waves. Data were statistically analyzed at each time point with a Student’s *t* test (*P* > 0.05 at each time point). In (H), for estradiol treatment, *n* = 46, and for DMSO treatment, *n* = 34. (A, D, E, G, and I) Calibration bars indicate fluorescence intensity in micrographs, from low (−) to high (+).

If the dynamics of local, vascular, and distal calcium waves all support the possibility of a passive transmission mechanism, then this brings models for active transmission of calcium waves into question. Focusing on the dual calcium/ROS wave ion channel–mediated propagation mechanism for salt-triggered calcium waves, which relies on a calcium-induced calcium release ion channel process involving the vacuolar channel TWO-PORE CHANNEL 1 (TPC1) and ROS production by the plasma membrane NADPH oxidase RESPIRATORY BURST OXIDASE HOMOLOGUE D (RBOHD) (*9*), we wounded *tpc1-2*, *rbohd*, and *rbohd/f* mutants expressing a calcium reporter. We found that in each mutant, wounding triggered a local wave with dynamics that matched those in wild-type plants (Fig. 3, A to C; fig. S4, A and B; and movie S2). Furthermore, wound-triggered vascular and distal waves occurred in each mutant with the same dynamics as those observed in wild-type plants (Fig. 3, D to F; fig. S4, C to E; and movie S3). Therefore, this calcium-induced calcium release/ROS wave mechanism does not drive wound-triggered calcium waves in *Arabidopsis* seedlings.

### Wound-triggered calcium waves do not rely on plasmodesmata

Previous studies observed that calcium waves in mutants with constitutively open or closed plasmodesmata were perturbed (*6*, *10*). Thus, these studies implicated the symplastic pathway (i.e., via the interconnected cytoplasm) in transmission of a mobile signaling molecule (chemical messenger) between cells. However, constitutive plasmodesmal closure impairs plant growth processes (*15*, *16*). To minimize these and other off-target effects of plasmodesmal closure, we generated plants that carry a calcium reporter and an inducible *LexA::icals3m* transgene that allows estradiol-triggered induction of callose deposition at plasmodesmata to reduce their aperture (*17*) and transiently perturbs the symplastic passage between cells (figs. S5 and S6). We wounded these plants 24 hours after estradiol or mock treatment, and the local, vascular, and distal calcium waves showed identical dynamics whether plasmodesmal closure was induced or not (Fig. 3, F to I; fig. S7, A to C; and movies S4 and S5). Thus, our data do not support a model in which wound-induced calcium waves are transmitted by a symplast-mobile chemical messenger.

### Local and distal wound–triggered calcium waves are dependent on GLR3.3

**Fig. 4.**
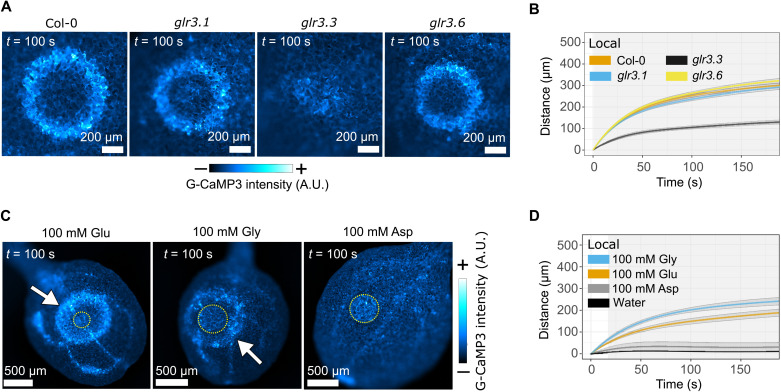
Local wound–induced calcium waves are dependent on *GLR3.3* and mimicked by application of amino acid agonists of GLRs. (**A**) Local wound–triggered calcium waves in glr3 mutants. (**B**) Mean distance (±SE) over time plots for the local wave peak progression in the glr3 mutants (Col-0, *n* = 18; glr3.1, *n* = 19; glr3.3, *n* = 20; and glr3.6, *n* = 20). Gray shading indicates when *P* < 0.01 for the comparison between the distance traveled by Col-0 and glr3.3 waves using Student’s *t* test at each time point. (**C**) Glutamate and glycine droplets (yellow circles) trigger local calcium waves (arrows), while aspartate does not. (**D**) Mean distance (±SE) over time plots for the wave peak progression of local calcium waves triggered by glutamate (*n* = 24), glycine (*n* = 9), aspartate (*n* = 6), and water (*n* = 5). The shaded area indicates when *P* < 0.05, comparing the distance traveled by the glutamate and glycine waves at each time point with Student’s *t* test. The distance traveled by both glutamate and glycine waves is greater than water at each time point with Student’s *t* test (*P* < 0.01), and the aspartate response shows no significant difference to the water control (*P* > 0.3 at all time points). (A and C) The calcium reporter is indicated for each panel, and calibration bars indicate fluorescence intensity in micrographs from low (−) to high (+).

Previous studies have shown that both GLR3.3 and GLR3.6 (*10*, *11*, *13*) are required for calcium wave transmission. GLRs are calcium-permeable channels activated by extracellular ligands and present a possible mechanism for translating a signal from an apoplastic mobile chemical messenger into an intracellular calcium influx. Wounding of *glr3.1* and *glr3.6* mutant plants triggered traveling local calcium waves with wild-type dynamics, but while some calcium response immediately surrounding the wound site was evident in the *glr3.3* mutant, the traveling waves were absent (Fig. 4, A and B, and movie S6). A previous study localized *GLR3.3* expression to the phloem, with GLR3.3-GFP (green fluorescent protein) localizing in endomembranes (*13*). However, recent single-cell transcriptomic data derived from *Arabidopsis* leaves identified *GLR3.3* transcripts in the mesophyll and epidermal cell populations, suggesting that expression occurs in a greater range of cell types (https://bioit3.irc.ugent.be/plant-sc-atlas/leaf) (*18*). Furthermore, using a transient, split GFP approach in *Nicotiana benthamiana*, we observed a pool of GLR3.3-GFP that localized to the plasma membrane (fig. S8), consistent with the topology and localization required to bind an extracellular ligand. Therefore, while it is possible that the reliance of calcium wave transmission on *GLR3.3* is via a non–cell autonomous mode of action or a pleiotropic effect induced by the mutation, the simplest interpretation of our data is that *GLR3.3* is expressed in the epidermis and mesophyll of cotyledons sufficiently to place GLR3.3 in the plasma membrane of these cells, where it is essential for the cell-to-cell transmission of calcium waves (i.e., local waves). This hypothesis infers that GLR amino acid agonists might be candidate mobile messengers that trigger cellular calcium responses.

### Amino acid agonists of GLRs trigger calcium waves

To explore the possibility that calcium waves are a direct readout of the mobility of GLR agonists, we investigated calcium responses to individual amino acids. We and others have observed that application of glutamate can trigger vascular and distal calcium waves (Fig. 1E). Thus, we applied exogenous drops of the GLR amino acid agonists glycine and glutamate (100 mM, pH 7) to cotyledons and observed that both glycine and glutamate, as well as a crude plant extract, elicited a local traveling calcium wave that slows with time (Fig. 4, C and D, and fig. S9). The nonagonist aspartate did not elicit a calcium wave (Fig. 4, C and D), and glutamate- and glycine-triggered waves were *GLR3.3* dependent (fig. S10). Quantitative analysis of droplet-triggered calcium waves shows that glycine triggers faster waves than glutamate (Fig. 4D) despite the GLR3.3 ligand binding domain having an almost twofold higher affinity for glutamate than glycine (*19*). As glycine is smaller than glutamate, the difference in local calcium wave speeds might indicate that dynamics depend on the size of the amino acid, consistent with the possibility that the waves are readout of diffusion of the agonist.

### Glutamate is released and diffuses from a wound site

An intensity-based glutamate-sensing fluorescent reporter (iGluSnFR) is commonly used in animals to report the presence of glutamate (*20*, *21*). We tested the specificity of iGluSnFR to glutamate in our system, applying glutamate and glycine to cotyledons of *35::CHB-iGluSnFR* transgenic plants that secrete iGluSnFR to the apoplast. In these experiments, glutamate triggered a radial wave of iGluSnFR fluorescence, but glycine did not trigger a significant response (fig. S11, A and B). Therefore, as glycine and glutamate trigger calcium waves of similar amplitude (Fig. 4C and fig. S12), these results support the conclusion that iGluSnFR fluoresces in the presence of specific amino acids and not because of the calcium wave or downstream responses such as apoplastic alkalinization. However, as apoplastic alkalinization carries the potential to shift the fluorescence of iGluSnFR (pKa 7.0 in the unbound form and 6.5 in the bound form) (*20*), we further verified that iGluSnFR fluorescence was not a product of pH changes associated with cellular responses. Thus, we performed a series of experiments in which we applied droplets of unbuffered solutions of 10 mM MgCl_2_ that ranged in pH 5 to 9 to mimic rapid physiological changes in pH arising from cellular release of molecules and ions. Unlike droplets of glutamate, none of these solutions induced a wave of iGluSnFR fluorescence 200 s after application (fig. S11, A and C). We also noted that neither 100 nor 200 mM NaCl triggered iGluSnFR fluorescence (fig. S11D), indicating that the glutamate-triggered response in cotyledons (also triggered by 50 mM glutamate; fig. S11E) is not a consequence of the osmotic potential of the solution. Therefore, apoplastic iGluSnFR is a reliable reporter for in vivo imaging of glutamate in plants.

**Fig. 5.**
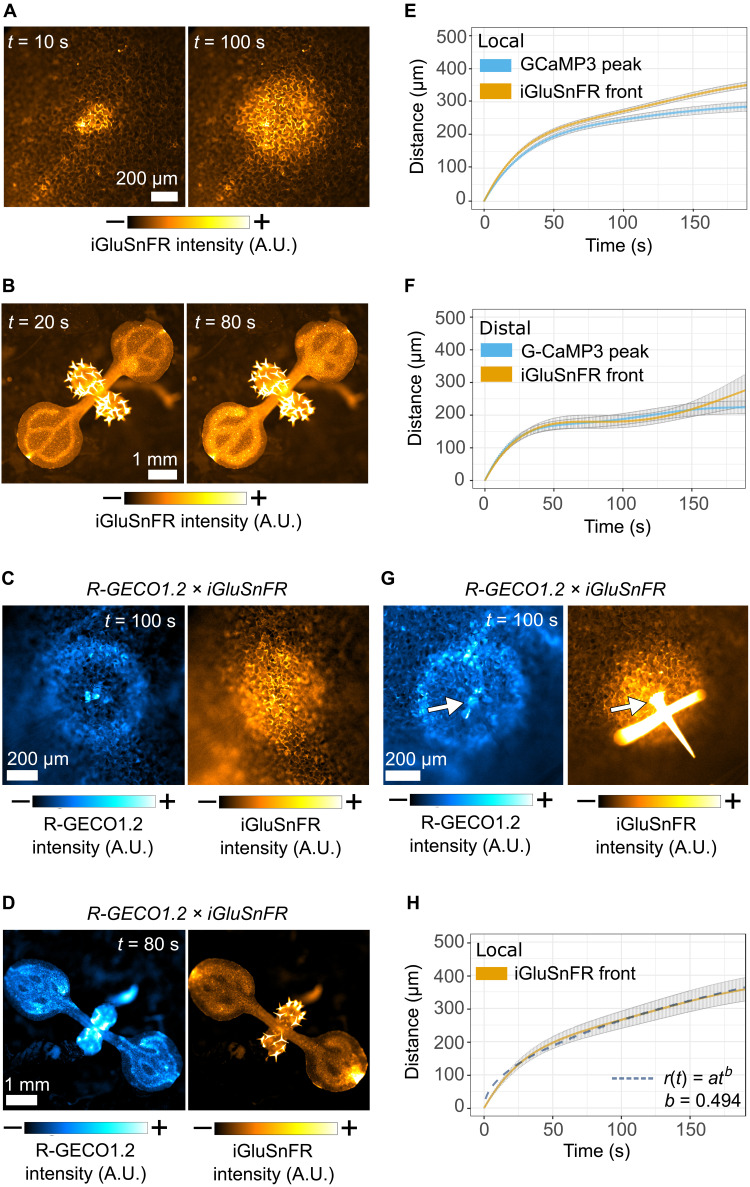
An apoplastic glutamate wave is coincident with wound- and touch-induced calcium waves. Micrographs of (**A**) local and (**B**) systemic glutamate responses to wounding. Micrographs of (**C**) local and (**D**) systemic calcium and glutamate responses to wounding in the iGluSnFR and R-GECO1.2 dual reporter. (**E**) Mean distance (±SE) over time plots of the local wound–triggered calcium wave peak and glutamate wave front in independent reporter lines (GCaMP3, *n* = 14 and iGluSnFR, *n* = 10). (**F**) Mean distance (±SE) over time plots of the distal calcium wave peak and glutamate wave front in independent reporter lines (G-CaMP3, *n* = 13 and iGluSnFR, *n* = 6). G-CaMP3 data are the same as shown in Fig. 1C. (**G**) Micrographs of local calcium and glutamate responses elicited by touch in the R-GECO1.2 and iGluSnFR dual reporter. (**H**) Mean distance (±SD) over time plot of the local wound–triggered glutamate wave (*n* = 11) with the fit of *r*(*t*) = *at^b^* to the data (dashed line). Calibration bars (A to C, D, and G) indicate fluorescence intensity in micrographs from low (−) to high (+).

To determine whether wounding triggers the release of glutamate, we wounded iGluSnFR reporter plants (Fig. 5, A and B) and observed the local and distal responses. Needle wounds induced a radially progressing front of fluorescence that emanates from the wound site and, similar to the calcium wave, slows with time. Similarly, stem cutting triggered systemic responses with a distal wave that emanates from the vasculature and slows with time. Progression of the local and distal calcium and glutamate waves in plants that carry both calcium (R-GECO1.2) and glutamate (iGluSnFR) reporters (Fig. 5, C and D) suggests that the glutamate wave front is coincident with the calcium wave peak. The coincidence of the wound-triggered calcium and glutamate waves was also evident in leaves of different ages, noting that when a systemic calcium wave was triggered in mature leaves, it was accompanied by a systemic glutamate wave (fig. S13). Quantitative analysis of the calcium and glutamate waves in independent reporter lines (Fig. 5, E and F) confirms that while the local glutamate front travels slightly further than the calcium wave, the calcium wave peak and the glutamate front in both local and distal responses travel with the same dynamics, supporting the possibility that both fluorescent signals are outputs of the same phenomenon. As further evidence that the mechanism for calcium wave transmission is common to multiple stimuli, touch triggers glutamate responses that, despite traveling slightly faster and further than their simultaneous calcium waves, also travel radially away from the stimulus site and slow with time (Fig. 5G, fig. S14, and movie S7).

To probe the mechanism by which the glutamate front might travel through tissue, we determined the time dependency of wound-triggered glutamate front by fitting the function *r*(*t*) = *at^b^*, in which *r*(*t*) is the distance from the origin to the front at time *t* and *a* and *b* are parameters that were allowed to vary freely to fit the data. The Einstein-Smoluchowski equation states that the square of the distance traveled by a diffusing molecule depends linearly on time, *r*(*t*)^2^ ∝ *t*, and thus, *r*(*t*) ∝ *t*^1/2^ (i.e., *b* = 0.5). Therefore, our finding that for the progression of the wound-triggered glutamate wave front, *b* = 0.494 ± 0.004 (Fig. 5H) indicates that these dynamics are consistent with diffusion. Furthermore, fitting a diffusion equation to the data gives a diffusion coefficient (*D*) of 116 μm^2^ s^−1^. As *D* is inversely proportional to molecular mass, this is consistent with the expected *D* for glutamate, approximately four times smaller than carboxyfluorescein diacetate for which *D* was experimentally defined in the apoplast of the *Arabidopsis* root cortex as ~34 μm ^2^ s^−1^ (*22*).

### Calcium waves alone do not trigger jasmonic acid responses

**Fig. 6.**
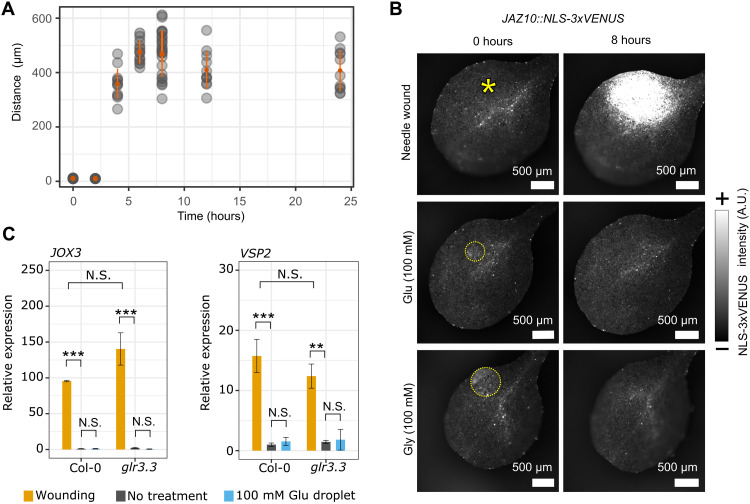
Wounding induces local JA responses, but amino acids do not. (**A**) Distance versus time plot for *JAZ10::nls-3xVenus* expression around a wound site (*n* = 23). Shading of data points indicates frequency of the observation, with darker points indicating more observations. The orange dot indicates the data mean, and the error bars indicate SEs. (**B**) Extended depth of focus micrograph of NLS-3xVenus fluorescence 0 and 8 hours after wounding and glutamate or glycine application in *JAZ10::nls-3xVenus* cotyledons. Deposited droplets are encircled by yellow dotted lines. Wound sites are indicated by a yellow asterisk. Calibration bar indicates the fluorescence intensity in micrographs from low (−) to high (+). (**C**) Reverse transcription quantitative polymerase chain reaction (RT-qPCR) data profiling relative expression of *JOX3* and *VSP2* in 10-day-old cotyledons of Col-0 and *glr3.3* upon different treatments 4 hours after treatment. Error bars represent SEs for three biological replicates. Each biological replicate contains 20 cotyledons from 10 seedlings. Statistical analysis was conducted via a linear mixed model, with Tukey adjustment for multiple comparisons. N.S. indicates *P* > 0.05; **P < 0.01 and ***P < 0.001. *JOX3*: Col-0 wound versus Col-0 no treatment, *P* = 3 × 10^−4^; Col-0 no treatment versus Col-0 100 mM glutamate droplet, *P* = 1; Col-0 wound versus *glr.3* wound, *P* = 0.052; *glr3.3* wound versus *glr3.3* no treatment, *P* < 1 × 10^−4^; and *glr3.3* no treatment versus *glr3.3* 100 mM glutamate droplet, *P* = 1. *VSP2*: Col-0 wound versus Col-0 no treatment, *P* = 5 × 10^−4^; Col-0 no treatment versus Col-0 100 mM glutamate droplet, *P* = 0.99; Col-0 wound versus *glr3.3* wound, *P* = 0.65; *glr3.3* wound versus *glr3.3* no treatment, *P* < 5 × 10^−3^; and *glr3.3* no treatment versus *glr3.3* 100 mM glutamate droplet, *P* = 1.

Our data strongly support a model in which calcium waves are a readout of the mobility of amino acids, but as systemic signaling is a complex phenomenon and wounding is a multicomponent stimulus comprising cellular damage, mechanical forces, and pressure changes, it is not clear whether all systemic responses are mediated by this transmission mechanism. Wounding triggers long distance electrical signals in addition to calcium waves, and both wounding and electrical stimuli can trigger jasmonic acid (JA) responses (*23*). Models also place calcium signaling upstream of JA responses (*7*), but it is not clear whether calcium waves can directly trigger JA signaling. To explore the relationship between calcium waves and JA signaling, we exploited our observation that amino acids can trigger calcium waves in the absence of wounding. Thus, we compared JA responses to wounding with those triggered by amino acid application. First, we wounded plants that express the JA response marker *JAZ10::nls-3xVenus* with a needle and observed that NLS-3xVenus was detected 4 hours after wounding in the area around the wound and spread to a maximum distance of ~500 μm from the wound site 8 hours after wounding (Fig. 6A). By contrast, localized application of neither glutamate nor glycine triggered expression of *JAZ10::nls-3xVenus* (Fig. 6B) despite their ability to trigger calcium waves (Fig. 4C). To broaden our survey of JA-associated responses, we investigated transcriptional changes of the JA-responsive genes *JOX3* (*24*, *25*) and *VPS2* (*26*) to wounding and glutamate in Col-0 and the *glr3.3* mutant and found that both *VPS2* and *JOX3* showed significant induction in response to wounding in both Col-0 and *glr3.3*, but not in response to application of glutamate droplets in either genotype (Fig. 6C). Therefore, our data suggest that calcium waves alone are insufficient to trigger JA responses.

## DISCUSSION

It is well established that plants rapidly transmit signals over long distances, carrying information between tissues and organs. In the absence of a nervous system, how a plant signals a rapid response to distal stresses must rely on unique mechanisms, but a comprehensive and integrated model that fully explains this phenomenon has not yet been constructed. Calcium waves are a key component of long-distance signaling in response to a variety of stresses, and here, we have used time-resolved quantification of calcium wave dynamics to probe the underlying processes and molecular machinery that drive them, allowing us to determine that not all wound-induced distal responses are transmitted by calcium signals alone.

Research into calcium wave transmission in plants has been propelled forward in recent years by the development of genetically encoded calcium reporters. However, most studies have relied on low-resolution analysis of calcium responses, quantifying increases in calcium-triggered fluorescence in a defined region of interest (often a whole leaf) as an indicator of wave characteristics (*6*, *7*, *10*, *13*). These total fluorescence measurements do not resolve the difference between a calcium wave response that passes through a region and a transient increase in calcium across the whole region. Furthermore, with respect to different calcium wave responses, they do not discriminate between changes in wave speed and amplitude. Therefore, such approaches do not offer the resolution required to analyze the spatiotemporal kinetics of calcium waves and the physical processes that drive them.

**Fig. 7.**
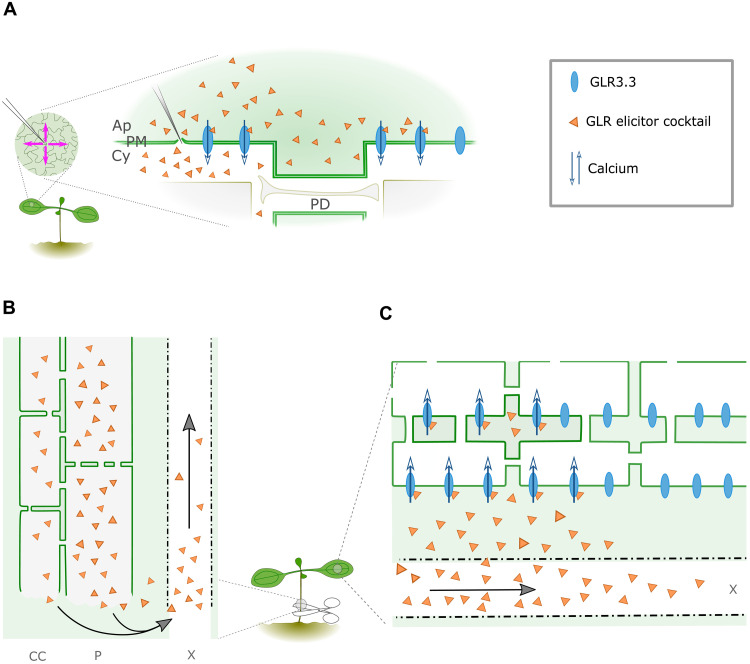
Model for local and systemic transmission of wound-induced calcium waves. (**A**) Cell-to-cell transmission of local calcium waves occurs by the release and diffusion of amino acid GLR agonists in the apoplast, activating GLRs that mediate intracellular increases in calcium as they travel. (**B**) When wounding severs a vein, as with stem or leaf cutting, a high volume of amino acids is released that travel rapidly through the xylem to distal leaves. (**C**) Amino acids are laterally released from the xylem and diffuse in the apoplast to mediate distal calcium waves. Ap, apoplast; PM, plasma membrane; Cy, cytoplasm; PD, plasmodesmata; CC, companion cells; P, phloem; X, xylem.

To better analyze the dynamics of fluorescence waves, we developed image analysis methods to track the peak or front of a fluorescence wave as it travels, enabling us to profile and quantify the dynamics of calcium waves elicited by a variety of stimuli. Using these methods in a variety of mutants and in response to a range of stimuli, we collected data that converge on a simple model in which calcium waves are transmitted by apoplastic diffusion and bulk flow of amino acids (Fig. 7). Specifically, our data are consistent with local calcium waves being a readout of diffusion of amino acids in the apoplast, with their limited range likely defined by the volume and concentration of amino acids released by the stimulus. Systemic waves are a combination of processes, with vascular calcium waves as readout of bulk flow of amino acids in the vasculature and distal waves as readout of diffusion of amino acids in the apoplast as they leave the vasculature (Fig. 7). We assume that as xylem vessels are continuous with the apoplast, the vascular pathway for amino acid flow is the xylem.

For amino acids to diffuse, or flow, from a site of stress, there must be a mechanism for their release into the apoplastic environment. In the example of wounding, release of amino acids likely occurs via the loss of membrane integrity that occurs during wounding and leakage of cell contents into the apoplastic space. However, in the case of touch or osmotic stress, how amino acids are rapidly released is less clear, but possibilities include that cellular stress responses involve the rapid activation of amino acid transporters, the exocytic release of amino acid containing vesicles as in neurotransmission in animals, or possibly by transient and nanoscale ruptures in the plasma membrane that allow leakage of cell contents (*27*, *28*). Having observed that living cells release amino acids that signal to neighboring cells (Fig. 5G), future research must investigate how cells release these and other signaling molecules in response to stress to understand intercellular and interorgan communication and response coordination in plants.

The ability of living cells to release amino acids, as occurs in touch or osmotic stress scenarios, also suggests the possibility that there is a multicomponent mechanism for the initiation of waves, i.e., signaling components exist that transmit stress perception to amino acid release. Further indicating the possibility of signaling complexity, a recent study implicated the stretch-activated anion channel MECHANOSENSITIVE CHANNEL OF SMALL CONDUCTANCE-LIKE 10 (MSL10) in both calcium and electrical responses to wounding (*7*). Unlike GLRs, MSL10 contributed to the amplitude and duration of these respective responses *(**7**)*, suggesting that additional mechanisms, including mechanoperception, regulate different components of systemic signaling. Given their association with the perception of mechanical forces, MSLs and other mechanoperception machinery (*29*–*33*) are possible candidates for signaling mediators of amino acid release, and responses such as the initial calcium burst observed when the *glr3.3* mutant is wounded (movie S6).

In addition to calcium waves, plants exhibit dynamic changes in membrane potential and cellular pressure that appear to travel away from a wound site, suggesting traveling electrical and pressure waves. While several models have been proposed to integrate these signals into a single mechanism (*6*, *7*, *28*, *34*), it is not clear how different signals relate to each other and whether they carry specific information. SWPs are stress-triggered electrical signals that are detected far from the site of stress. SWPs are likely related to calcium waves, as they follow the same path as calcium waves through the plant (*13*), are tuned by calcium availability in the apoplast (*35*), initiate coincidentally with calcium responses (*7*, *13*, *36*), and rely on GLR3.3- and GLR3.6-like calcium responses (*13*). Furthermore, like we propose here for calcium waves, SWPs have also been proposed to be mediated by a mobile chemical messenger (*37*, *38*). However, despite these similarities, the specific relationship between the two signals remains unresolved. A key distal response to stress is the induction of JA signaling, and a previous study showed that electrical stimulation of tissue is sufficient to elicit JA production and responses (*22*). Our data indicate that amino acid–mediated calcium waves cannot trigger JA responses, which suggests the possibility that electrical and calcium signals might elicit independent responses. While our finding conflicts with a previous observation that glutamate triggers the expression of JA-responsive genes (*10*), we note that these experiments were performed with a wounding component that our results suggest would independently trigger JA responses. The complexity of stimuli and signaling components illustrates the need to analyze the responses to each signal independently to better illustrate the phenomenon of systemic signaling in plants.

The mechanism that drives the transmission of calcium waves among cells, tissues, and organs has long been enigmatic. Here, we have demonstrated that calcium waves have dynamics consistent with being driven by bulk flow and diffusion of amino acids, establishing this as a viable model for calcium wave transmission. While this model simplifies our understanding of calcium wave transmission, systemic signaling incorporates multiple signals and responses, and chemical messengers are likely only part of a greater suite of processes. However, these data lay out new questions for the field regarding the identity and range of chemical messengers that carry signals through plants, the stimuli, and mechanisms that release them and the information that they carry.

## MATERIALS AND METHODS

### Plant materials and growth

The mutants and reporter lines used in this study are detailed in table S1. All transgenes and mutations are in *Arabidopsis thaliana* Col-0 ecotype. Seeds were stratified in sterile water for 3 to 4 days in the dark at 4°C and germinated on soil. Plants were grown in controlled cabinets (Panasonic Versatile Environmental Test Chamber MLR-352-PE) equipped with six Newlec light-emitting diode (LED) T8 color temperature at 4000 K and nine Newlec LED T8 color temperature at 6500 K (10-hour light/14-hour dark at 22°C). Nine- to 10-day-old seedlings were used to study local responses, and 11- to 13-day-old seedlings were used to study systemic responses.

### Generation of genetic material

Lines generated in this study were made by crossing existing transgenic lines or by transformation of *A. thaliana* Col-0 using the floral dip method (*39*) with *Agrobacterium tumefaciens* GV3101.

### Generation of DNA constructs

The pL2-1 binary vector carrying the *35S::R-GECO1.2* sequence was donated by G. Oldroyd. The binary vector containing the *LexA::icals3m* sequence was assembled via Golden Gate cloning, inserting polymerase chain reaction (PCR)–amplified *icals3m* sequence downstream of the PCR-amplified *LexA* promoter sequence into the backbone pICSL86933OD (containing the *nos::NPTII::Ocs* selection cassette and *Act2::XVE::mas* cassette).

### Wound, agonist, and touch stimulation

Needles for wounding or for delivering droplets were pulled from Drummond glass capillaries (microcaps, 50 μl; catalog no. 1-000-0500) with a Narishige PE-2 needle puller (magnet setting, 5.15; heater setting, 4.35). For wounding, needles were mounted on a custom-assembled micromanipulator mounted on a scissor jack platform. Systemic calcium waves were elicited by cutting the tip of a cotyledon with 5-mm spring-loaded scissors (FTS 91500-09) in the presence or absence of a droplet of liquid on the cotyledon surface, the stem of a seedling, or the petiole of leaf 1 in adult plants. When performing cuts in the presence of a droplet of liquid, the droplet was placed on the intact surface of a cotyledon, and the cut was performed by cutting through the droplet.

Needles for delivery of droplets were coated in VALAP (lanoline:paraffin:petroleum jelly, 1:1:1) to create a hydrophobic surface that allowed for efficient delivery of droplet on the cotyledons. After coating, the needle tip was opened by gently touching the tip on a petri dish surface. Needles were then front-loaded by dipping the open tip in the required solution and unloaded on the leaf using a rubber bulb mounted at the back end of the capillary. For elicitation of local responses, droplets of elicitors were placed on the surface of intact cotyledons. For salt- and glutamate-triggered systemic waves, droplets were applied to the edge of a cut cotyledon. For HPTS experiments, droplets of 0.4 mM HPTS were placed on an intact cotyledon, and the cotyledon was cut in the droplet. Uncoated needles mounted on the micromanipulator were also used for trichome bending, i.e., needles were held parallel to the surface of true leaves and brushed across it to stimulate one or multiple trichomes on a single leaf.

### Time course imaging

For time-lapse imaging, we used an Axio ZoomV16 stereomicroscope, equipped with a SPECTRA Light Engine (Lumencor) light source, a monochrome digital camera ORCA-FLASH4 (C13440, Hamamatsu), and a Zeiss Plan Z 1×/0.25 objective. In all experiments, seedlings were imaged in growth pots. R-GECO1.2 was excited at 525 to 558 nm with a light source power of 10%, and fluorescence was collected with a 605/70-nm band-pass filter and an exposure time of 250 ms. G-CaMP3 and iGluSnFR were excited at 463 to 487 nm with a light source power of 3%, and fluorescence was collected with a 525/50-nm band-pass filter and an exposure time of 370 ms. For R-GECO1.2, GCaMP3 and iGluSnFR frames were collected every 2 s with a size of 1024 × 1024 pixels, 16-bit depth, and 2 × 2 binning. Venus was excited at 503 to 519 nm with a light source power of 10%, and fluorescence was collected with a 544/24-nm band-pass filter, 370 ms of exposure with a size of 1024 × 1024 pixels, 16-bit depth, and 2 × 2 binning. Venus images of whole cotyledons were collected as *Z* stacks composed of a set of 15 optical sections, centered at the site of stimulus application and with a *Z* step of 20 μm. The Zeiss Blue software (version 2.6) built-in function “extended depth of focus” (with the default method “wavelets”) was used to create *z* stack projections. For time course imaging of 3-week-old plants, the Zeiss Plan Apo ×0.5 objective was used, with a 463- to 487-nm light source (60% power) and a 525- to 558-nm light source (60% power) with a 370-ms exposure for R-GECO1.2 collection and 500-ms exposure for iGluSnFR collection.

### Image analysis for cotyledon experiments

Image analysis was performed in Fiji (*40*). To study the cell-to-cell progression of responses over time, the fluorescence signal was assessed at successive time points for each time series. For local calcium waves, glutamate waves, and *JAZ10::NLS-3xVENUS*, individual time point images were analyzed by scanning fluorescence along a series of centered radii using Fiji macros developed for the purpose (fig. S1, A to F). For analysis of local wounding responses, 100 scanning radii depart from the wound site (code is available at https://doi.org/10.5281/zenodo.6584998). For trichome bending or droplet-induced responses, three points were defined at the edge of the trichome base or at the edge of the droplet, which defined a circle that anchors a series of 700-μm scanning radii between two of the points (fig. S1E; code is available at https://doi.org/10.5281/zenodo.6585004).

To analyze distal calcium or glutamate waves (i.e., the waves that travel out from the veins in the unelicited cotyledon), we defined a 200-μm-long vein segment and generated 10 equidistant 500-μm perpendicular scanning transects that depart from the segment (fig. S1G; code is available at https://doi.org/10.5281/zenodo.6585014). In all these cases, the fluorescence intensity profile at each pixel position along each radius or transect at each time point was measured for data analysis. To analyze images of vascular waves, the distance between the wound site and the front of the vascular wave over time was measured manually by tracing the wave at each time point in Fiji.

### Data analysis for cotyledon experiments

All data were analyzed using R (*41*). For analysis of local and distal signals, the average intensity profile across all radii at each time point was calculated, and a curve built with the nonparametric local regression method “loess” (locally estimated scatterplot smoothing) fit on the profile at each time point. Functions of the Zoo package (*42*) were used to detect the local maxima of the fitted loess curve, retaining their distance from the center and intensity. The local maxima that represented the peak of the wave were identified on the basis of its intensity and on the expectation that its distance would increase from the start site at successive time points.

Baseline fluorescence levels were calculated as the average signal intensity in the tissue before it was reached by the response, while noise levels around the baseline were estimated as

$$\text{noise}=\text{baseline}\pm 1.96\times \bar{\text{SD}}$$

where $\bar{\text{SD}}$ is the average SD of the average intensity across the whole dataset. For each data point, the ratio between the intensity of the detected peak and the baseline upper noise value was calculated and used to estimate the signal/baseline noise ratio of the detected peak as an indicative measure of the intensity of the response. This is visualized as different shades of blue for each data point in the wave profile plots.

The front of the wave was defined as the point where the average fluorescence signal profile reaches a threshold defined as

$$\text{threshold}=\text{baseline}+\bar{\text{SD}}$$

For local wounds, the time point 0 s was set to the frame where the needle hits the surface of the cotyledon. For the distal signals and droplet-induced signals, time point 0 s was set to the moment when the peak or the front of the wave initially progresses forward from the vein or the edge of the droplet.

The position of the wave peak and/or the wave front over time were summarized by fitting a polynomial of degree 6 that passes through the origin of the axes to the data of each replicate. The average polynomial across all replicates was then used to describe the position of the wave peak and/or wave front over time. The first derivative of this polynomial was used to describe the velocity of the wave peak and/or wave front over time. The average progression of the signal for different genotypes or conditions was compared using Student’s *t* test at each time point. The R code for the analysis of the local waves in response to needle wounding is available at https://doi.org/10.5281/zenodo.6584998. The R code for the analysis of the local waves in response to elicitors is available at https://doi.org/10.5281/zenodo.6585004. The R code for the analysis of distal waves is available at https://doi.org/10.5281/zenodo.6585014.

To study the progression of vascular waves, the measured distances that the front of the calcium wave reached along the vasculature from the wound site over time were plotted against the time. A straight line passing from the origin of the axes was then fit on these data points for each replicate. The velocity of the wave in each replicate was defined by the slope of the line, and data from different conditions and genotypes were compared using the Wilcoxon rank sum test (code is available at https://doi.org/10.5281/zenodo.6585016).

### Data modeling

Curve fitting was carried out in R, using the “lm” function to fit linear models and the “nls” function for nonlinear regression. The initial parameters in the optimization of the distance over time curves (*r*(*t*) = *kt^b^* and $r(t)=\sqrt{6\mathrm{Dt}}$) were *b* = 0.8 and *D* = 60 μm^2^/s (https://doi.org/10.5281/zenodo.6585006). For vascular wave analysis, we fit a GP model to the manually measured distances over time for HPTS and the calcium wave (R-GECO fluorescence) using the R function GauPro. To estimate the effect of Taylor dispersion of a diffusing molecule in the xylem that triggers a calcium wave, we used equations 12 and 13 from (*4*) with the following parameters: flow speed = 500 μm/s, diffusion constant = 10^−6^ cm^2^/s, and xylem radius = 10 μm and fit the channel activation threshold parameter. As this equation was derived for a constant speed flow speed, we restricted analysis to the time points up to 10 s for which this condition approximately holds.

### Preparation of amino acid solutions

Glycine (G/0800/60, Fisher Chemical), l-glutamic acid monosodium (49621, Sigma-Aldrich), and l-aspartic acid (A9256, Sigma-Aldrich) were dissolved in sterile deionized water to reach a concentration of 100 mM. For all solutions, pH was adjusted to 7 using 0.1 M NaOH if necessary.

### Preparation of crude seedling extract

Eight- to 10-day-old soil grown Col-0 seedlings were harvested and frozen in liquid nitrogen. Seedlings were finely ground using an electric pestle while still frozen. Samples were thawed and centrifuged for 15 min at 15,871*g* at 4°C. The supernatant was collected and centrifuged again, with the collected supernatant forming the “crude extract” used in experiments. The extract was used immediately.

### Estradiol treatment

Twenty-four hours before experiments, *LexA::icals3m* plants were treated with 20 μM β-estradiol (Sigma-Aldrich) in 0.1% dimethyl sulfoxide (DMSO) solution or 0.1% DMSO solution (control solution). Both solutions were diluted in deionized sterile water with 0.01% Silwet L-77 (De Sangosse). The solutions were applied to seedlings using a hobby airbrush (Silverline, 380158), while sink leaves in adult plants were painted with brushes.

### Callose staining and quantification

A solution of 0.1% aniline blue (415049, Sigma-Aldrich) in 1× phosphate-buffered saline (pH 7.4) was used to stain callose at plasmodesmata. Cotyledons, detached from soil-grown 8- to 10-day-old seedlings, were infiltrated with aniline blue by gently pressing a coverslip on a cotyledon immersed in the staining solution. The cotyledons were then rinsed in sterile water before mounting on a microscope slide. Images of the upper epidermis were acquired on a Zeiss LSM 800 confocal microscope using a water immersion ×63 objective [C-Apochromat 63×/1.20WKorr ultraviolet-visible-infrared (UV-vis-IR) water]. Aniline blue was excited with a 405-nm laser at 2% power, and fluorescence was collected in the range 410 to 470 nm. *Z* stacks were acquired in two regions of each cotyledon, with 8 to 10 slices for each stack and an interval between slices of 0.3 μm. Twenty cotyledons were analyzed for the untreated *LexA::icals3m* line, and 18 cotyledons were analyzed for the estradiol-treated line. Image size was 1024 × 1024 pixels, with a 16-bit depth and 4× line averaging. Aniline blue signal at plasmodesmata was automatically quantified in Fiji using a macro developed in this work. Data output of this analysis were analyzed in R to obtain a summary of the mean integrated density of the detected particles in each *z* stack (the mean intensity of aniline blue staining signal per particle in each *z* stack, which correlates with the levels of callose at plasmodesmata). Fiji macro and R code for this analysis are available at https://doi.org/10.5281/zenodo.6583765.

### Evaluation of cytoplasmic connectivity in cotyledons via microprojectile bombardment

Microprojectile bombardment was used to evaluate cell-to-cell connectivity levels by quantifying enhanced GFP spread from a transformed cell into the neighboring untransformed ones as in previous studies (*43*). The Bio-Rad biolistic PDS-1000/He particle delivery system (*44*) was used to transform epidermal cells with the plasmid pB7FWG2.0. To do so, 1.2 mg of 1-nm gold particles (Bio-Rad) were coated with 5 μg of the required plasmid DNA by precipitation with 1.25 M CaCl_2_ and 15 mM spermidine, resuspended in 100 μl of 100% ethanol, and then shot at cotyledons at 1350 psi. To prepare seedlings for bombardment, 2 to 3 hours before bombardment, 8- to 10-day-old *Arabidopsis* soil-grown seedlings were transferred to petri dishes containing 0.8% agar-MS medium (the hypocotyl was held with tweezers and gently pushed into the agar so that the cotyledons were resting on the surface of the agar). Cotyledons were imaged for data collection 18 to 30 hours after bombardment.

Images were acquired on a Zeiss LSM 800 confocal microscope using EC Plan-Neofluar 10×/0.30 M27-air objective. Samples were excited with a 488-nm laser at 1% power, and emission was collected in the range 505 to 546 nm. *Z* stacks were acquired to cover the entire *Z* range necessary to count the number of epidermal cells that showed GFP signal. Counting of the number of cells that showed GFP signal was carried out in Fiji. The transformed cell was counted as 0, and every other epidermal cell showing GFP signal and in contact with the transformed cell or with GFP-showing neighbors was counted as an additional 1. After the blind counting procedure, data were reconnected to the corresponding genotype, and the statistical analysis was carried out in R using a bootstrap approach (*45*).

### Evaluation of cytoplasmic connectivity in adult leaves via 5-carboxyfluorescein diacetate phloem loading

The four youngest leaves of 5-week-old *LexA::icals3m Arabidopsis* plants were painted with estradiol or DMSO control solution on their abaxial and adaxial side. Twenty-four hours after treatment, droplets of 1 mM CFDA (5-carboxyfluorescein diacetate; Sigma-Aldrich, C4916) were placed on expanded source leaves, and the leaves were wrapped in cling film to avoid evaporation of the solution and improve uptake. Four to 5 hours after CFDA loading, the abaxial surface of the third youngest leaf was imaged using a Axio ZoomV16 stereomicroscope, equipped with a SPECTRA Light Engine (Lumencor) light source, a monochrome digital camera ORCA FLASH4 (C13440, Hamamatsu), and a Zeiss Plan Z ×1.5/0.25 objective at ×66 to ×67 zoom. One to two *Z* stack images were taken for each leaf in the lower half of the leaf, with 3-μm *z* steps, illuminating the sample with 463- to 487-nm light and collecting fluorescence with a 525/50-nm band-pass filter and 50-ms exposure. The Zeiss Blue software (version 2.6) built-in function extended depth of focus (with the default method wavelets) was used to create *z* stack projections.

Image analysis was performed in Fiji (*17*) using a macro developed for the purpose that scanned each image by 50 vertical and 50 horizontal equidistant lines (fig. S6) and recorded the intensity profile of the image along each line. Data were analyzed in R (*32*) using a peaks and valleys detection algorithm. A nonparametric local regression method loess was used to fit a curve on the intensity data along each grid line. Exploiting this curve, functions of the Zoo package (*33*) were used to detect local maxima and minima. The positions of the left and right base of each peak were defined as the point where the curve was higher than the minimum intensity of the neighboring valley + 2× SD of residuals. The width of a peak was calculated as the distance between the position of the left and right base of that peak and acts as a measure for the distance traveled by carboxyfluorescein (CF) out from the veins. All the widths of all the peaks across all the grid lines of a *z* stack projection were averaged to obtain one mean peak width value for each *z* stack projection. Fourteen and 16 z stack projections were analyzed for estradiol- and DMSO-treated plants, respectively, with samples belonging to a total of nine estradiol-treated plants and 11 DMSO-treated plants. The Fiji and R code used for this analysis are available at https://doi.org/10.5281/zenodo.6585010.

### Localization of GLR3.3-GFP in *N. benthamiana*

*GLR3.3* was cloned from guide DNA; the stop codon was removed, and central Bsa I and Bb SI restriction sites were domesticated via PCR without altering the amino acid sequence. The *GLR3.3* sequence was fused to a *Myc-GFP11* fragment, with 35*S* promoter and terminator sequences by Golden Gate cloning in a level 1 plant expression vector. The resulting plasmid (FP01159) was transformed into *A. tumefaciens* GV3101 and coinfiltrated into *N. benthamiana* leaves with *A. tumefaciens* GV3101 carrying the PEP101E plasmid (Addgene) that encodes for in planta expression of *GFP1-10.* Material for imaging was harvested 2 or 3days after infiltration. Leaves were infiltrated with 16 μM FM 4-64 (Thermo Fisher Scientific) before imaging. Images were acquired on a Zeiss LSM 800 confocal microscope using water dipping ×63 objective (C-Apochromat 63×/1.20WKorr UV-vis-IR water). GFP was excited at 488 nm with an argon laser, and emission was collected at 500 to 545 nm. FM 4-64 was excited at 561 nm with a diode pumped solid state (DPSS) laser, and emission was collected at 590 to 620 nm.

### Reverse transcription quantitative PCR

Ten-day-old soil-grown Col-0 UBI10::G-CaMP3 and *glr3.3* UBI10::G-CaMP3 seedlings were wounded and treated with 100 mM glutamate droplets or left untreated. For wounding, the cotyledon was supported by a cotton swab and two wounds (one on either side of the main vein) were performed using a stainless steel acupuncture needle [Seirin, J-type, no.02(0.12) × 30 mm]. Glutamate droplets were applied as described above. Four hours after treatment, cotyledons were harvested and frozen in liquid nitrogen. Twenty cotyledons, belonging to 10 seedlings were harvested for each biological replicate of each treatment for each genotype. Three biological replicates were analyzed. RNA was extracted (RNeasy Plant Mini kit, QIAGEN), deoxyribonuclease treatment (TURBO DNA-free kit, Invitrogen) and cDNA synthesized with the High-Capacity cDNA Reverse Transcription kit (Thermo Fisher Scientific). Reverse transcription quantitative PCR (RT-qPCR) on *JOX3* (At3g55970) and *VSP2* (At5g24770) using *UBC21* (At5g25760) as housekeeping gene was performed with a LightCycler 480 (Roche) using LightCycler 480 SYBR Green I Master (Roche). Primers used are listed in table S2. Data analysis was carried out according to Hellemans *et al.* (*46*). Statistical analysis was carried out on the relative normalized quantities of the three biological replicates using a linear mixed model and the R package lme4 (*47*). *P* values were corrected for multiple comparisons using Tukey method in the R package emmeans (*48*).

### Data visualization

All plots were generated in R. Box plot features follow the default R settings: The upper boundary of the box is the 75th percentile (or third quartile); the line in the middle of the box is the median (50th percentile or second quartile), and the lower boundary of the box is the 25th percentile (first quartile). The distance between the upper and lower boundary of the box is the interquartile range (IQR). The whiskers extend to the most extreme data point, which is no more than 1.5× IQR away from the upper boundary (upper whisker) or lower boundary (lower whisker). The “*X*” inside the box plots indicates the mean. The violin plots are realized with the R “ggviolin” function (from the package “ggpubr”). The violin plots show the kernel probability density of the data at different values, i.e., the wider a section of the violin, the higher the probability of the data of that group to fall there. Micrographs shown in the same panel and showing the same fluorescence channel are adjusted to the same levels of brightness/contrast unless otherwise specified.

### Quantification and statistical analysis

The data collected for each experiment is described in Materials and Methods. All data analysis was performed in R. Data were statistically analyzed by the following tests: the distance traveled by a calcium wave at each time point (Figs. 3, A to C and H, and 4, A and D, and figs. S1D; S4, B to D; and S7, A and B), Student’s *t* test; vascular wave speed (Figs. 2A and 3F), Wilcoxon rank sum test; number of cells showing GFP by microprojectile bombardment (fig. S5C), bootstrapping; intensity of aniline blue stained callose (fig. S5A), Wilcoxon rank sum test; and RT-qPCR, linear mixed model and Tukey *P* value adjustment. *N* and *P* values are indicated in the figure legends.

## Supplementary Materials

### This PDF file includes:

Figs. S1 to S14 Tables S1 and S2 References

## Notes

View/request a protocol for this paper from *Bio-protocol*.
