# Supplementary material for: Diffusion and bulk flow of amino acids mediate calcium waves in plants
Source: Sci Adv. 2022 Oct 21;8(42):eabo6693. doi: 10.1126/sciadv.abo6693 (PMC9586480; doi:10.1126/sciadv.abo6693)
Supplement: Supplementary file 1 — Figs. S1 to S14 Tables S1 and S2 References [file sciadv.abo6693_sm.pdf]

Science Advances
AAAS

# Supplementary Materials for

## Diffusion and bulk flow of amino acids mediate calcium waves in plants

Annalisa Bellandi *et al.*

Corresponding author: Christine Faulkner, christine.faulkner@jic.ac.uk

*Sci. Adv.* **8**, eabo6693 (2022)
DOI: 10.1126/sciadv.abo6693

**The PDF file includes:**

Figs. S1 to S14
Tables S1 and S2
Legends for movies S1 to S7
References

**Other Supplementary Material for this manuscript includes the following:**

Movies S1 to S7

**Figure S1.**

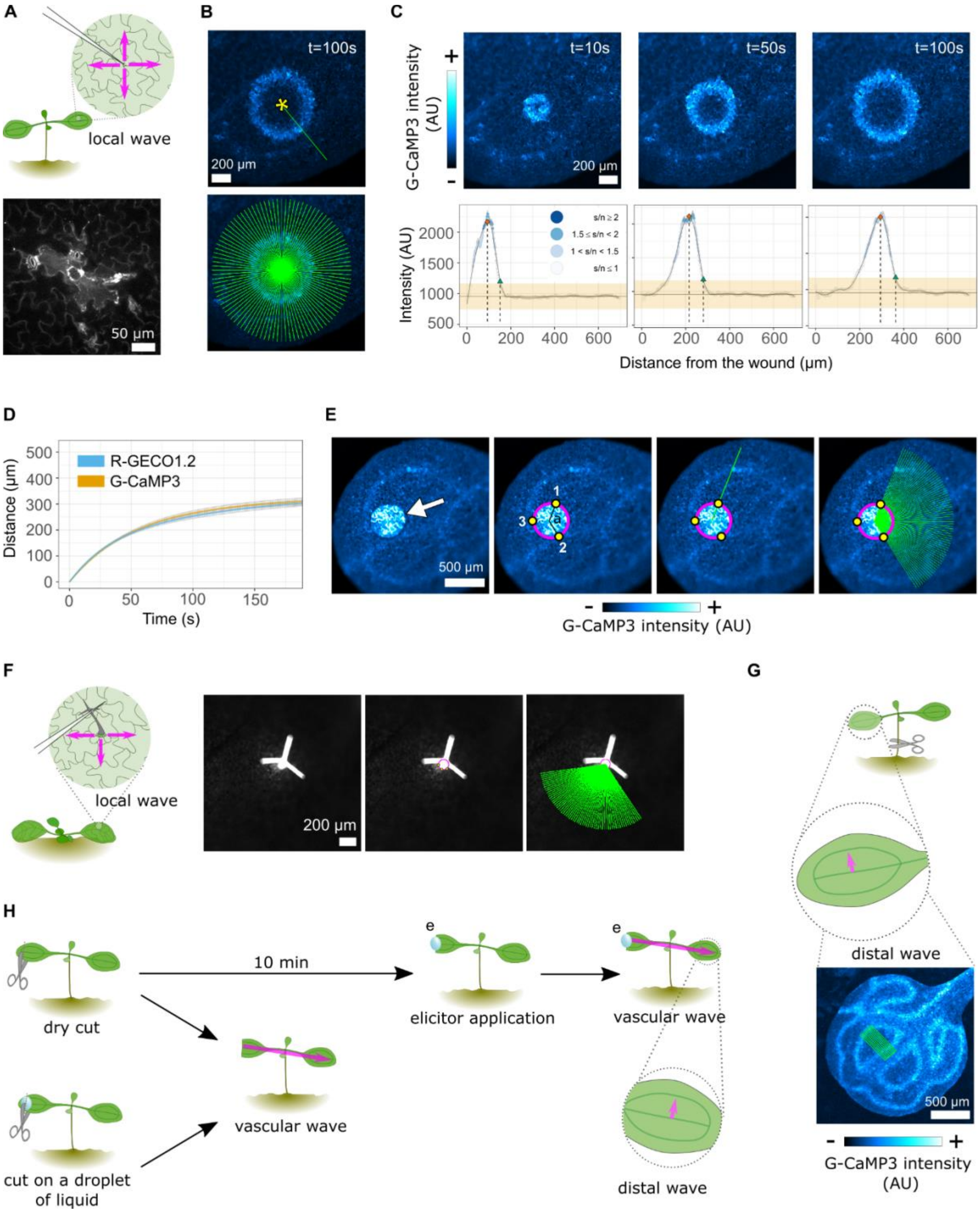
A
local wave
50 µm
B
t=100s
200 µm
C
G-CaMP3 intensity (AU)
+
-
t=10s
t=50s
t=100s
200 µm
s/n ≥ 2
1.5 ≤ s/n < 2
1 < s/n < 1.5
s/n ≤ 1
Intensity (AU)
Distance from the wound (µm)
D
R-GECO1.2
G-CaMP3
Distance (µm)
Time (s)
E
500 µm
G-CaMP3 intensity (AU)
F
local wave
200 µm
G
distal wave
500 µm
G-CaMP3 intensity (AU)
H
dry cut
10 min
elicitor application
vascular wave
vascular wave
distal wave
cut on a droplet of liquid

**Figure S1: Methods for the elicitation and analysis of local, vascular and distal calcium waves:** (**A**) Method for the elicitation of local calcium waves by needle wounding in cotyledons and the entity of the damage caused by this technique, visualized by propidium iodide staining and maximum projection of confocal optical sections (lower image). (**B**) Micrograph of the calcium wave 100 s post wound. The wave is scanned by 100 x 700 μm radii (lower image) that originate from the wounding site (yellow asterisk, top image). (**C**) Example of wave profiling result at t= 10, 50 and 100 s. Data points represent the average fluorescence intensity across all radii at each time point after wounding, with error bars representing SE across all radii. A Loess (locally estimated scatterplot smoothing) curve is superimposed on the data and describes the profile of the wave. The orange diamond marks the peak of the wave, the green triangle marks the front of the wave, the horizontal black line marks the baseline fluorescence intensity, the yellow area is baseline +/- average SD across the whole dataset as explained in the methods, the top of the yellow area marks the threshold for the detection of the front of the wave. Colouring of each data point represents the ratio between the fluorescence intensity of that datapoint and the upper limit of the noise level around the baseline, as defined in the methods. (**D**) Distance (± SE) over time for progression of the wound-triggered calcium wave peak in Col-0 seedlings that produce G-CaMP3 or R-GECO1.2. The distance travelled by the wave peaks in R-GECO1.2 and G-CaMP3 seedlings was compared at each timepoint by a Student's t-test, $p > 0.01$ at all timepoints. (**E**) Micrographs and analysis method for responses elicited by deposition of an elicitor droplet on the surface of a cotyledon. White arrow indicates the droplet. Three points (1, 2, 3) are marked on the edge of the droplet and define a circle (magenta) from the centre of which the scanning radii will originate. Equidistant scanning radii are generated between point 1 and 2, spanning the intermediate angle α. (**F**) Diagram representing the method of trichome touching using a glass needle and the method for scanning the response. The scanning method follows the same steps as in E, after generating a circle that marks the edge of the trichome base. (**G**) Diagram representing the stem-cutting method to elicit vascular and distal waves with a representative micrograph of the distal wave response. Distal wave progression was analysed along perpendicular transects (green) that start at the main vein as indicated on the micrograph. (**H**) Diagram representing the cotyledon tip-cutting method for the study of the vascular and distal waves. The tip of a cotyledon is cut with scissors to remove approximately 1/3 of the cotyledon, in absence or presence of a droplet of liquid on the surface of the cotyledon. This was primarily used to study the progression of vascular calcium waves. To study vascular and distal waves in response to an elicitor, a droplet of elicitor (e) was applied on the cut edge of the cotyledon after 10 min recovery period.

**Figure S2**

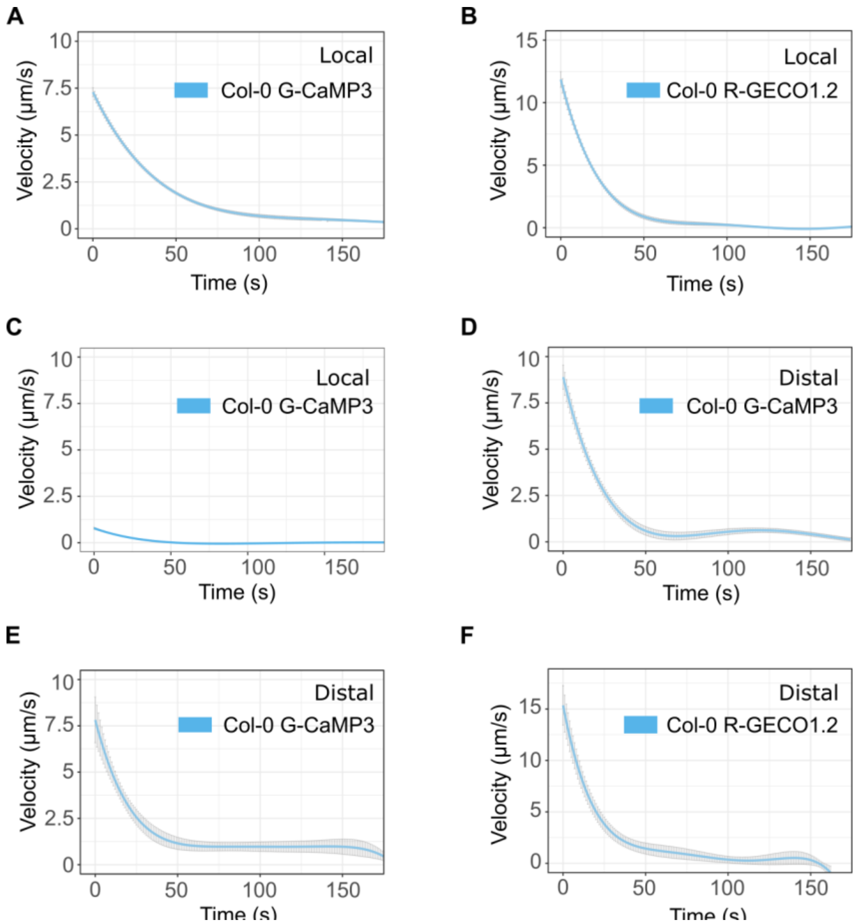


**Figure S2: Velocity of local and distal calcium waves in Figure 1 triggered by different stimuli:** Velocity (± SE) over time of local calcium waves upon (**A**) wounding (n = 11) and (**B**) touch (n = 13) and (**C**) 200 mM NaCl (n = 6). Velocity (± SE) over time for distal calcium waves triggered by (**D**) stem cut (n = 13), (**E**) 100 mM Glutamate (n = 10) and (**F**) 200 mM NaCl (n = 6). Data are derived from data in Figure 1.

**Figure S3**

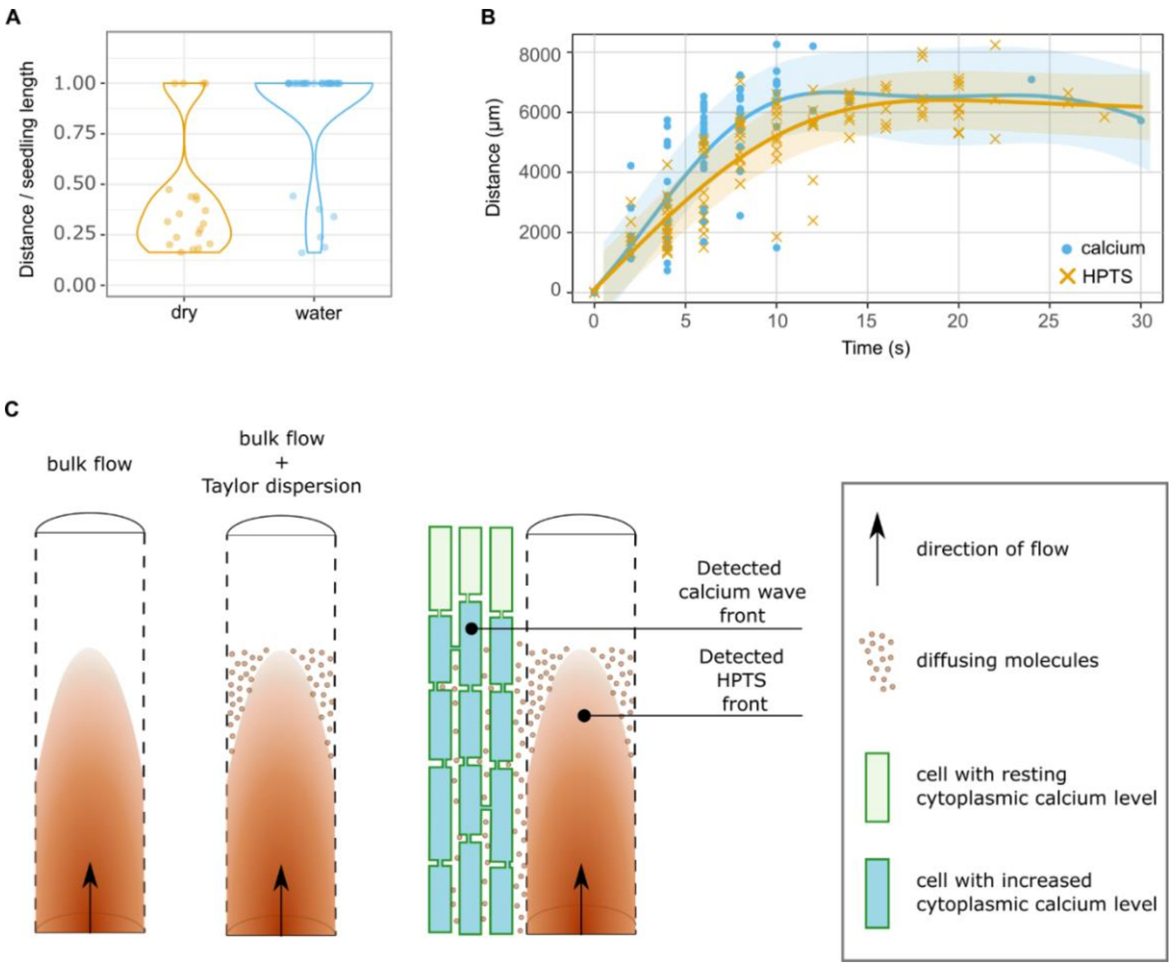


**Figure S3: Dynamics and modelling of wound-induced vascular calcium waves:** (**A**) Violin dot plots of the distance reached by vascular calcium waves induced by cutting the tip of a cotyledon in a droplet of water or dry. The distance reached by the calcium wave divided by the maximum length of the possible systemic path (from cut edge to end of the main vein in the opposite cotyledon). (**B**) Raw data for the progression of calcium waves and the HPTS signal along the vasculature in R-GECO1.2 seedlings upon cutting the tip of a cotyledon in an HPTS droplet. Gaussian process curves (± SE) are indicated by the orange (HPTS) and blue (calcium) lines. (**C**) Cartoon representing bulk flow with Taylor dispersion. Bulk flow transports fluid through a pipe with a shear effect that induces a cone-shaped distribution of fluid (left). At the surface of the cone, diffusion occurs that releases molecules ahead of the bulk flow (middle). In a tissue context (right) these diffusing molecules can diffuse out from the flow path and activate responses in adjacent cells, allowing them to be detected ahead of the molecules in the bulk flow

**Figure S4**

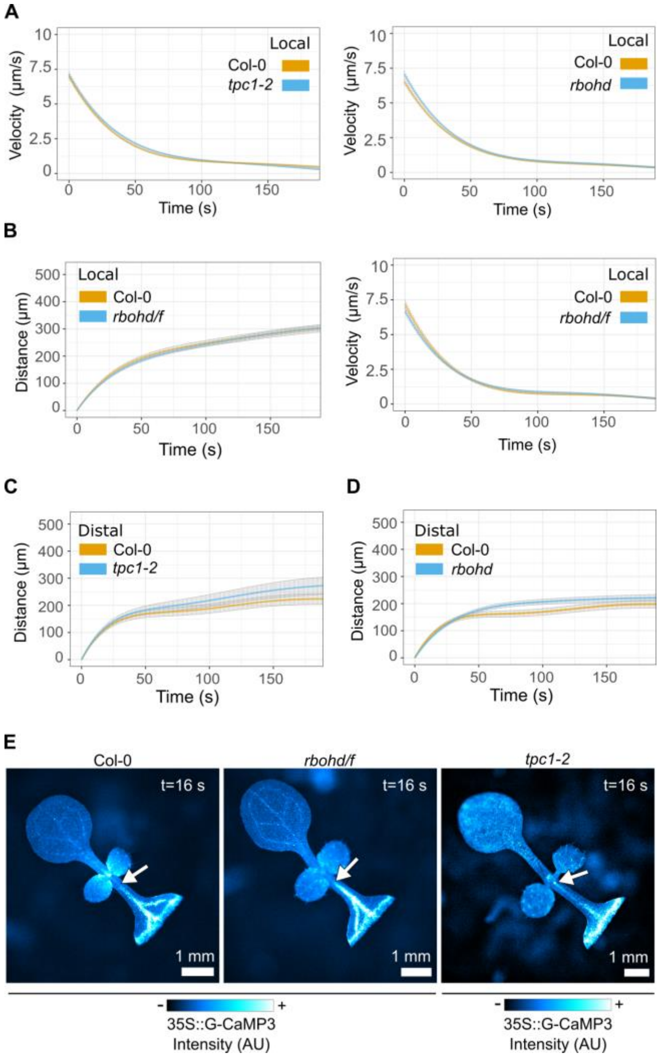


**Figure S4: Quantification of local and distal waves and images for vascular waves in *rbohd*, *rbohd/f* and *tpc1-2*:** (**A**) Mean velocity (±SE) over time of local wound-induced calcium waves shown in Fig 3B, C for Col-0, *tpc1-2* and *rbohd* mutants. (**B**) Mean distance (±SE) over time and velocity (±SE) over time for local wound-triggered calcium waves in the *rbohd/f* mutant (n = 23) compared to Col-0 (n = 26). (**C-D**) Mean distance (±SE) over time for the stem-cut triggered distal calcium wave in *tpc1-2* (n = 13) and *rbohd* (n = 15) mutants relative to Col-0 (n = 13 and n = 14 respectively). Col-0 data in C is the same shown in 1 C. Data was analysed by a Students' t-test (A-D) at each timepoint, $p > 0.01$ for all timepoints. (**E**) Micrographs of vascular calcium waves elicited by cutting the tip of a cotyledon in Col-0, *rbohd/f* and *tpc1-2* mutants. White arrows indicate the front of the detected vascular calcium wave. Colour bar indicates fluorescence intensity from low (-) to high (+). Brightness/Contrast of *tpc1-2* image differentially adjusted compared to Col-0 and *rbohd/f* images.

**Figure S5**

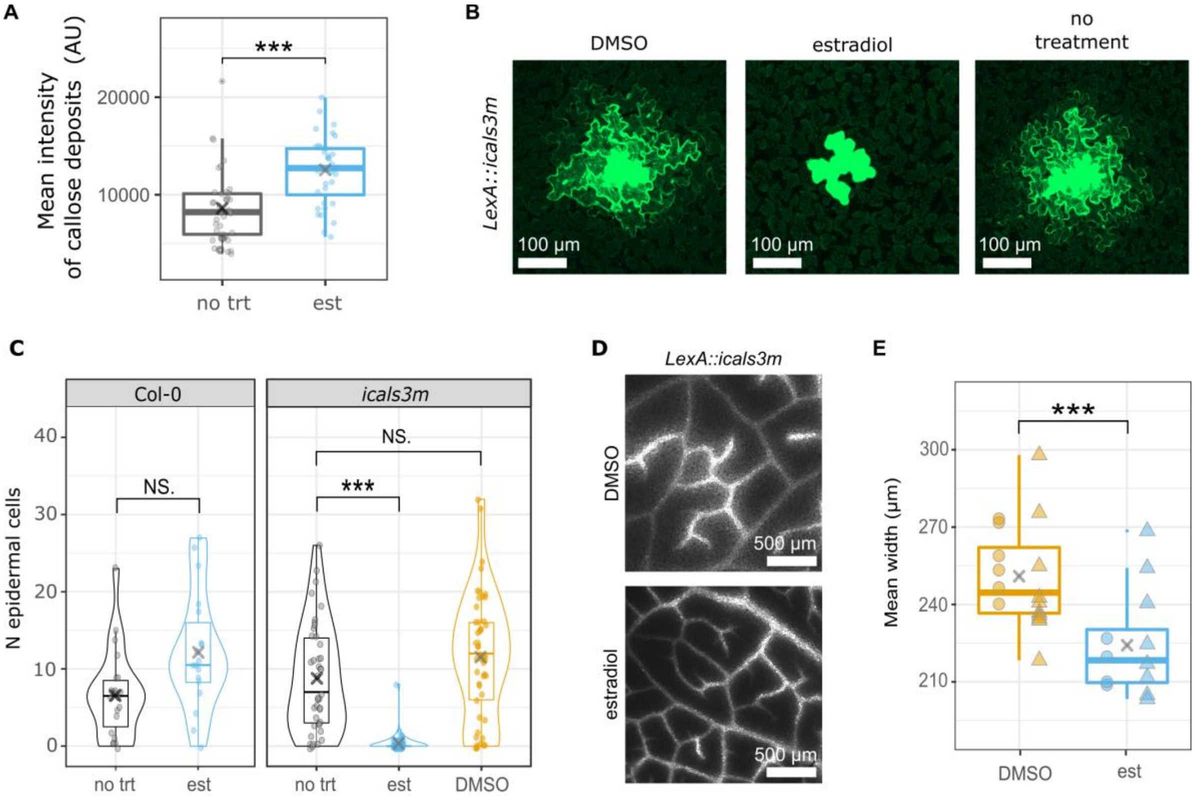


**Figure S5: *LexA::icals3m* allows estradiol-triggered plasmodesmal callose deposition and closure.** (A) Mean callose intensity per plasmodesma per image in induced (est, n = 36 images) and uninduced (no trt, n = 40 images) *LexA::icals3m* cotyledons. Statistical test is Wilcoxon Rank-sum test, *** indicates $p < 0.001$ ($p = 6.7 \times 10^{-6}$). (B) Maximum projections of confocal z-stacks of microprojectile bombardment sites in untreated and mock (DMSO) treated *LexA::icals3m*, and induced (estradiol) *LexA::icals3m* cotyledons. In induced *LexA::icals3m* (estradiol), GFP is seen in fewer neighbouring cells indicating plasmodesmata are closed. (C) Quantification of the number of cells showing GFP in each treatment condition for Col-0 (no trt n = 22, est n = 18) and *LexA::icals3m* (no trt n = 41, est n = 50, DMSO n = 49). Estradiol treatment of *LexA::icals3m* seedlings induces a significant reduction in the spread of GFP to surrounding cells. Statistical comparison by bootstrapping, *** indicates $p < 0.001$, NS indicates $p > 0.05$ (Col-0 estradiol vs no treatment $p = 0.059$, *LexA::icals3m* estradiol vs no treatment $p = 5.09 \times 10^{-4}$, *LexA::icals3m* no treatment vs DMSO $p = 0.074$). (D) Extended depth of focus micrographs of CF fluorescence in un-induced (DMSO) and induced (estradiol) *LexA::icals3m* sink leaves, ~4-5 hours after loading CFDA on source leaves. (B) Quantification of CF spread from the vasculature in induced and un-induced *LexA::icals3m* leaves. Statistical test is Wilcoxon Rank-sum test, *** indicates $p < 0.001$ ($p = 4.2 \times 10^{-4}$). Estradiol n = 14, DMSO n = 16. The shape of the data points indicates data points belonging to the same experiment.

**Figure S6**

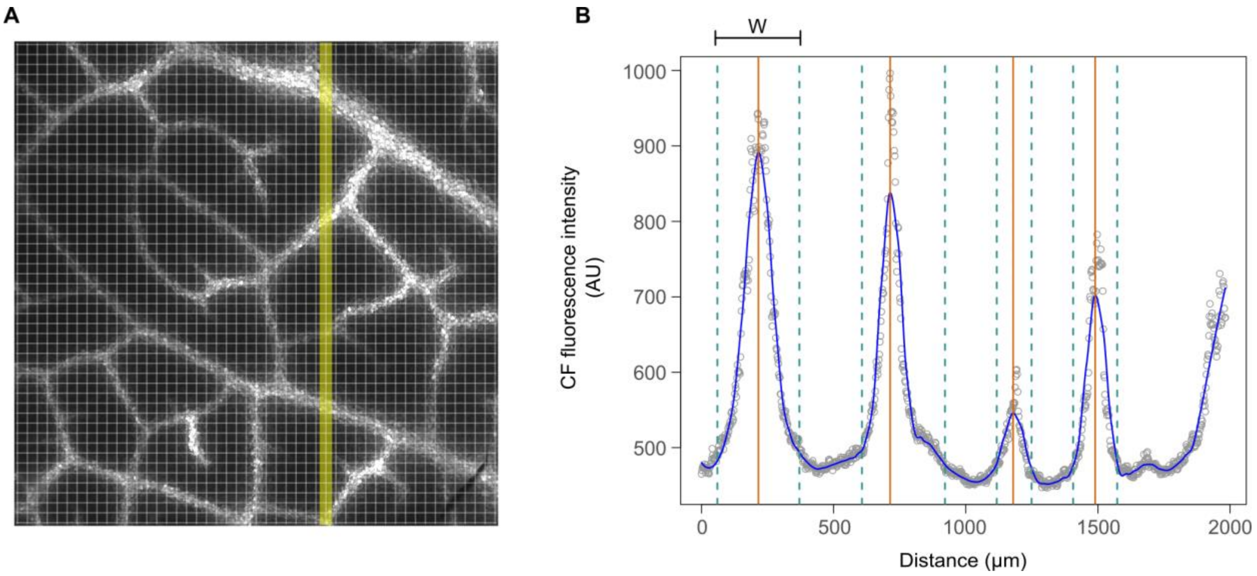


**Figure S6: Quantification method for the CFDA assay**. (**A**) Each extended depth of focus micrograph is scanned along 50 vertical and 50 horizontal equidistant lines arranged in a grid. The grid is here overlayed to the image presented in S5D. The fluorescence intensity along each grid line is then analysed as in B. (**B**) Profile of fluorescence intensity along the grid line outlined in yellow in A. The grey dots represent the fluorescence intensity values along the grid line obtained from the image, the superimposed solid blue line is the loess (locally estimated scatterplot smoothing) curve that describes the fluorescence profile along the gird line. Detected peaks of fluorescence intensity corresponding to the vasculature are marked by orange solid vertical lines, the detected left and right base of the peaks are marked by dotted green lines. For each peak, the width (W) is the distance between the left and right base. The average width of all peaks across all the grid lines of one image constitute one data point in S5E.

**Figure S7**

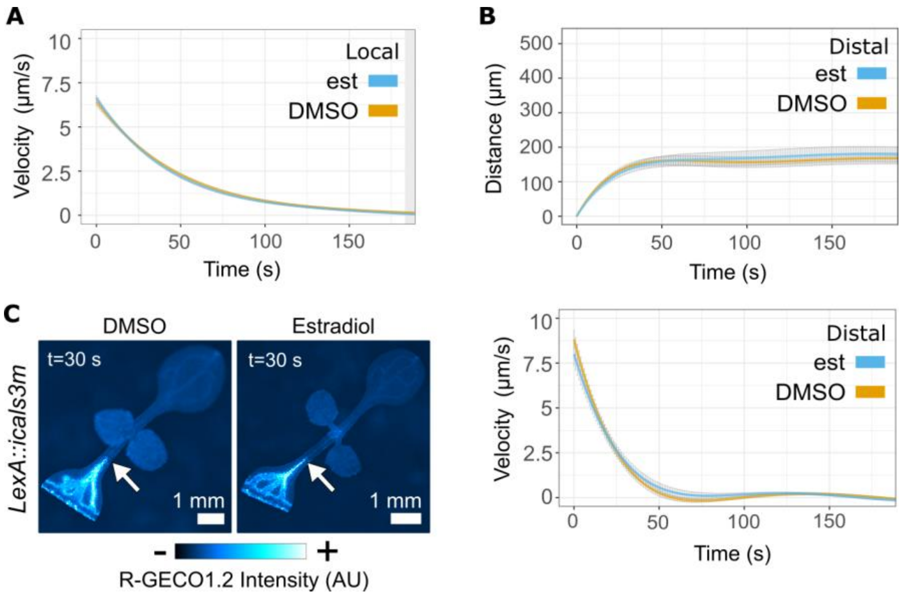


**Figure S7: Plasmodesmata closure does not impair transmission of calcium waves.** (**A**) Mean velocity (± SE) over time plots for the progression of the local calcium wave peak in *LexA::icals3m* mock treated (DMSO) and induced (est), from data in Fig. 3E. (**B**) Mean distance (± SE) over time plots for the progression of the distal calcium wave peak in *LexA::icals3m* mock treated (DMSO, n = 13) and induced (est, n = 11). Statistical test in A and B is Students' t-test, $p < 0.01$ is indicated by the grey shaded area. (**C**) Micrographs of vascular calcium waves elicited by cutting the tip of a cotyledon in mock treated (DMSO) and induced (estradiol) *LexA::icals3m*. White arrows point to the front of the vascular wave. The colour bar indicates fluorescence intensity from low (-) to high (+).

**Figure S8**

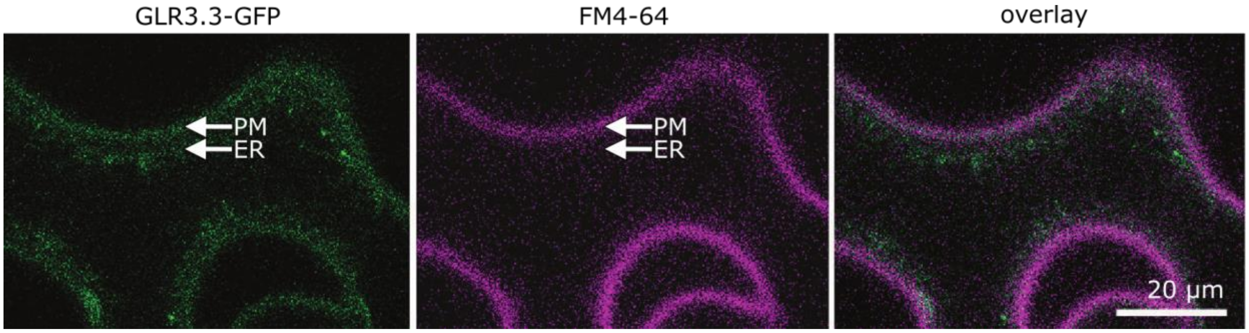


**Figure S8: Subcellular localization of GLR3.3-GFP.** Confocal micrograph of a *N. benthamiana* epidermal cell transiently expressing *GLR3.3-GFP* and stained with FM4-64. GLR3.3-GFP (left), FM4-64 (centre) and overlay of the two channels (right). The overlay shows overlap of the GLR3.3-GFP signal and FM4-64 signal at the plasma membrane.

**Figure S9**

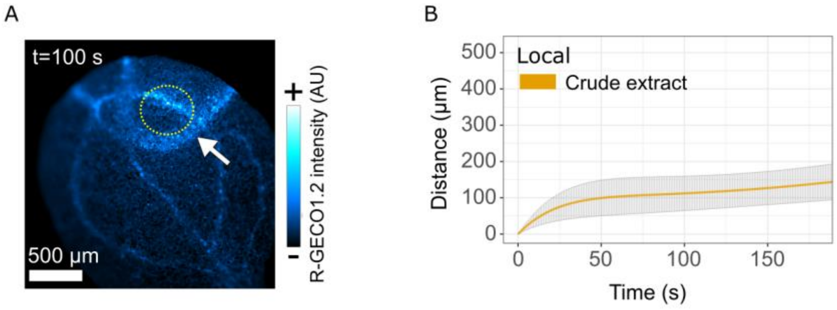


**Figure S9: Crude extract from seedlings induces a calcium wave that slows with time.** (**A**) Micrograph showing calcium wave (white arrow) triggered by application of a droplet of crude seedling extract (yellow circle). (**B**) Mean distance (± SE) over time plots of the local calcium wave peak triggered by application of a droplet of crude extract (n = 6).

**Figure S10**

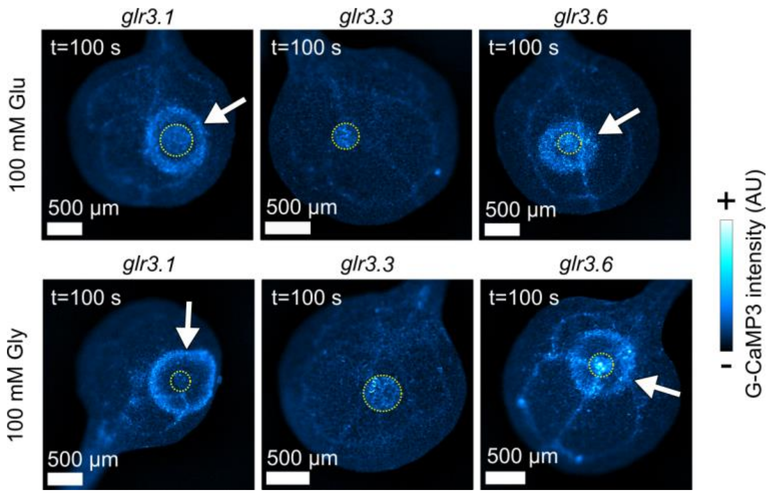


**Figure S10: Amino acid triggered calcium waves are *GLR3.3* dependent**. Micrographs showing calcium waves (white arrows) triggered by application of droplets of glutamate and glycine on *glr3.1*, *glr3.3* and *glr3.6* mutants. Waves are triggered in *glr3.1* and *glr3.6* mutants, but not in the *glr3.3* mutant. Droplets are marked by yellow dashed circles.

**Figure S11**

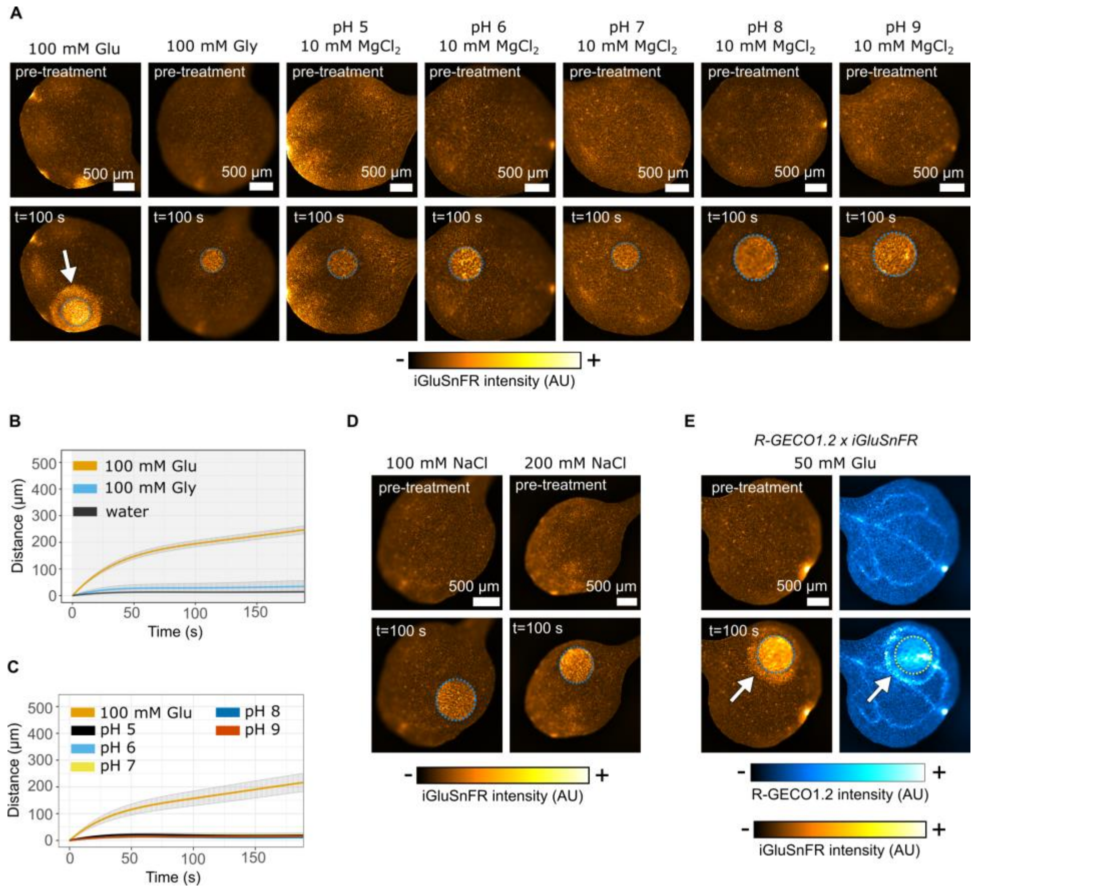


**Figure S11: iGluSnFR reports the presence of glutamate**. (**A**) Exogenous drops of glutamate but not glycine trigger a radial wave of iGluSnFR fluorescence. (**B**) Distance travelled by the wave front (±SE) over time plots for amino acid triggered waves of iGluSnFR fluorescence. Water n = 6, glutamate n = 11, glycine n = 8. The shaded region indicates where $p < 0.01$ when comparing the glutamate response to the water control (Students' t-test at each time point). The same analysis of the response between the water control and the glycine droplets indicates no statistical difference ($p > 0.05$ at all timepoints). (**C**) Distance travelled by the wave front (±SE) over time plots for waves of iGluSnFR fluorescence triggered by solutions at 10 mM $MgCl_2$ pH 5, 6, 7, 8, 9. 100 mM Glu n = 7, pH 5 n = 10, pH 6 n = 10, pH 7 n = 11, pH 8 n = 11, pH 9 n = 11. Statistical comparison (Students' t-test at each time point) between response elicited by 10 mM $MgCl_2$ pH 5 and the other 10 mM $MgCl_2$ solutions indicates no statistical difference ($p > 0.05$). (**D**) Micrographs before and 100s after deposition of exogenous drops of 100 mM NaCl or 200 mM NaCl. NaCl droplets don't trigger a radial wave of iGluSnFR fluorescence. (**E**) Micrographs of a cotyledon of the iGluSnFR and R-GECO1.2 dual reporter before and 100 s after deposition of 50 mM glutamate droplets. 50 mM Glutamate droplets elicit calcium and glutamate radial waves of fluorescence. In A, D and E calibration bar indicates intensity of fluorescence from low (-) to high (+). Droplets are marked by a blue or yellow dashed circle, white arrow indicates increase of iGluSnFR or RGECO1.2 fluorescence around the 100 mM glutamate or 50 mM glutamate droplet.

**Figure S12**

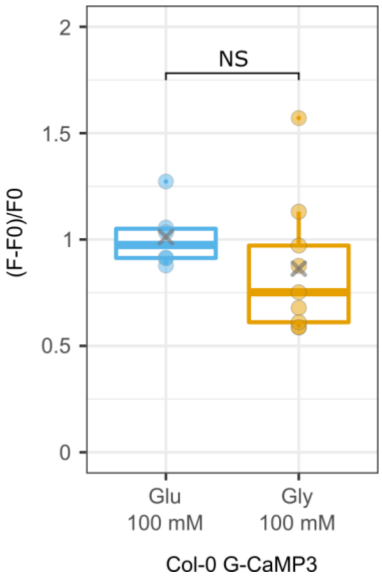


**Figure S12: Comparison between the amplitude of calcium waves elicited by 100 mM glutamate or 100 mM glycine.** Statistical test is Wilcoxon Rank-sum test, NS indicates $p > 0.05$ ($p = 0.14$). F is the maximum fluorescence intensity reached by the wave profile, F0 is the average baseline fluorescence intensity in the tissue at resting state. Glutamate n = 6, Glycine n = 9.

## Figure S13

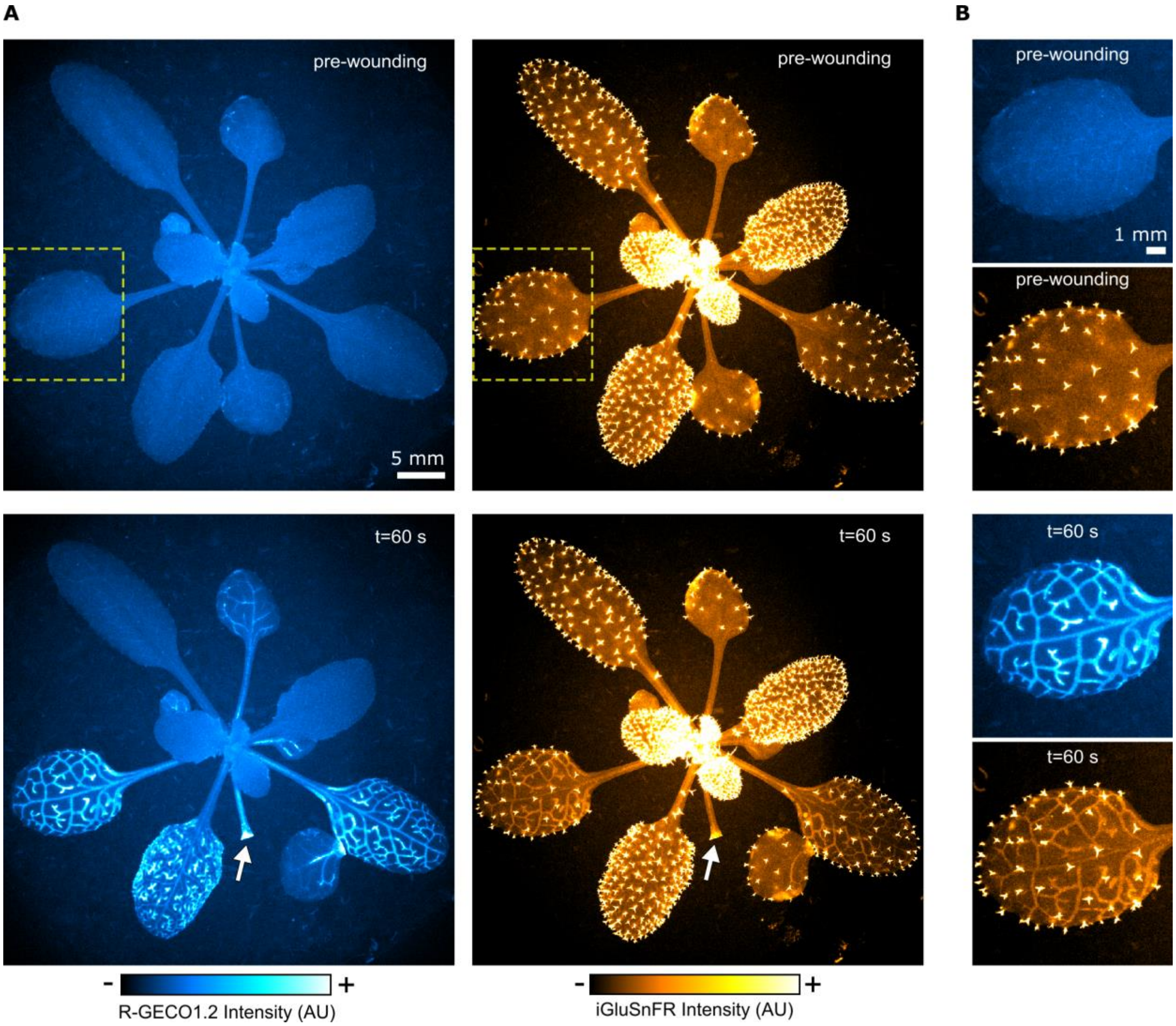


**Figure S13**: Coincidence of calcium and glutamate systemic signals in adult plants. (A) Micrograph of 3-week old iGluSnFR and R-GECO1.2 dual reporter plant before wounding and after leaf 1 was excised with a pair of scissors. White arrow indicates the cutting site. Calibration bar indicates intensity of fluorescence from low (-) to high (+). (B) Magnification of the region indicated by a yellow dashed rectangle (leaf 3) in A.

**Figure S14**

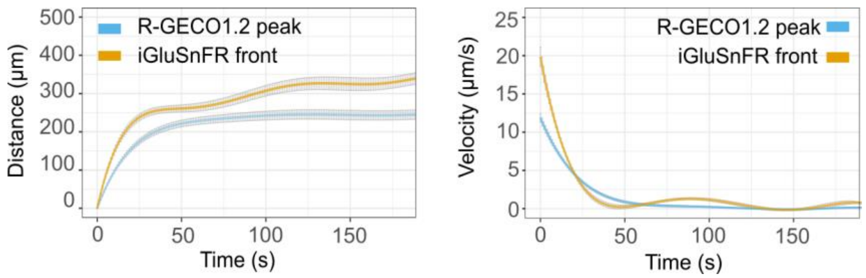


**Figure S14: Quantification of calcium and glutamate waves upon trichome touch.** Mean distance (±SE) and velocity (±SE) over time plots for the progression of the local calcium wave peak and the glutamate wave front following trichome touch in the iGluSnFR and R-GECO1.2 dual reporter plants. The R-GEC01.2 curve is derived from the same data as presented in 1B.

**Table S1:** List of mutants and reporter lines used in this study

| Line | Gene ID | Reference |
|---|---|---|
| *35S::R-GECO1.2* | - | this study |
| *35S::GCaMP3* | - | (11) |
| *rbohd x 35S::GCaMP3* | AT5G47910 | this study |
| *rbohd/f x 35S::GCaMP3* | AT5G47910/ AT1G64060 | this study |
| *tpc1-2 x 35S::GCaMP3* | AT4G03560 | (11) |
| *pUBI10::GCaMP3* | - | (13) |
| *glr3.1 x UBI10::GCaMP3* | AT2G17260 | (13) |
| *glr3.3 x UBI10::GCaMP3* | AT1G42540 | (13) |
| *glr3.6 x UBI10::GCaMP3* | AT3G51480 | (13) |
| *LexA::icals3m* | - | this study |
| *35S::CHIB-iGluSnFR* | - | (10) |
| *LexA::icals3m x 35s::R-GECO1.2* | - | this study |
| *35S::CHIB-iGluSnFR x 35s::R-GECO1.2* | - | this study |
| *JAZ10::NLS-3xVENUS* in Col-0 | - | (40) |
| *JAZ10::NLS-3xVENUS* in *aos* | - | (40) |

**Table S2:** List of primers used for RT-qPCR

| Primer name | Primer sequence | Gene of interest | Reference |
|---|---|---|---|
| JOX3_F | GGGTGACCAAATTCAGATGCTGAG | *JOX3* (At3g55970) | (41) |
| JOX3_R | AGGAACATTGCCCTTTGGGTTG | *JOX3* (At3g55970) | (41) |
| VSP2_F | CATCATAGAGCTCGGGATTGAACCC | *VSP2* (At5g24770) | (41) |
| VSP2_R | AGATGCTTCCAGTAGGTCACGC | *VSP2* (At5g24770) | (41) |
| AyB36 | GACGCTTCAGTCTGTGTGTAGAGC | UBC21 (At5g25760) | - |
| AyB37 | CTTAGAAGATTCCCTGAGTCGCAG | UBC21 (At5g25760) | - |

**Supplementary Movie Legends**

**Movie S1:** HPTS and calcium vascular waves upon cutting the tip of a R-GECO1.2 cotyledon on a droplet of HPTS.

**Movie S2**: The local calcium response to needle wound in Col-0, *rbohd*, *rbohd/f* and *tpc1-2* mutants. Reporter is G-CaMP3.

**Movie S3:** The vascular and distal calcium response to cotyledon tip cutting in Col-0, *rbohd/f* and *tpc1-2* mutants. Reporter is G-CaMP3**.**

**Movie S4:** The local calcium response to needle wounding in un-induced and induced *LexA::icals3m x R-GECO1.2* line

**Movie S5:** The systemic (vascular and distal) calcium response to stem cutting in un-induced and induced *LexA::icals3m x R-GECO1.2* line

**Movie S6:** The local calcium response to needle wounding in cotyledons of Col-0, *glr3.1*, *glr3.3* and *glr3.6* mutants. Reporter is G-CaMP3.

**Movie S7:** The calcium and glutamate response upon trichome touch in the iGluSnFR and R-GECO1.2 dual reporter line.

## REFRENCES AND NOTES


1. D. D. Cunningham, The causes of fluctuations in turgescence in the motor organs of leaves; and, a new and parasitic species of *Choanephora*. *Ann. Roy. Bot. Gard. Calcutta* **6**, (1895).

2. U. Ricca, Transmission of stimuli in plants. *Nature* **117**, 654–655 (1926).

3. A. L. Houwink, The conduction of excitation in *Mimosa pudica*. *Recueil des travaux botaniques neerlandais* **32**, 51–91 (1935).

4. M. G. Blyth, R. J. Morris, Shear-enhanced dispersion of a wound substance as a candidate mechanism for variation potential transmission. *Front. Plant Sci.* **10**, 1393 (2019).

5. E. E. Farmer, D. Gasperini, I. F. Acosta, The squeeze cell hypothesis for the activation of jasmonate synthesis in response to wounding. *New Phytol.* **204**, 282–288 (2014).

6. Y. Fichman, R. Mittler, Integration of electric, calcium, reactive oxygen species and hydraulic signals during rapid systemic signaling in plants. *Plant J.* **107**, 7–20 (2021).

7. J. Moe-Lange, N. M. Gappel, M. Machado, M. M. Wudick, C. S. A. Sies, S. N. Schott-Verdugo, M. Bonus, S. Mishra, T. Hartwig, M. Bezrutczyk, D. Basu, E. E. Farmer, H. Gohlke, A. Malkovskiy, E. S. Haswell, M. J. Lercher, D. W. Ehrhardt, W. B. Frommer, T. J. Kleist, Interdependence of a mechanosensitive anion channel and glutamate receptors in distal wound signaling. *Sci. Adv.* **7**, eabg4298 (2021).

8. W. G. Choi, G. Miller, I. Wallace, J. Harper, R. Mittler, S. Gilroy, Orchestrating rapid long-distance signaling in plants with $Ca^{2+}$ , ROS and electrical signals. *Plant J* **90**, 698–707 (2017).

9. M. J. Evans, W. G. Choi, S. Gilroy, R. J. Morris, A ROS-assisted calcium wave dependent on the AtRBOHD NADPH oxidase and TPC1 cation channel propagates the systemic response to salt stress. *Plant Physiol.* **171**, 1771–1784 (2016).

10. M. Toyota, D. Spencer, S. Sawai-Toyota, W. Jiaqi, T. Zhang, A. J. Koo, G. A. Howe, S. Gilroy, Glutamate triggers long-distance, calcium-based plant defense signaling. *Science* **361**, 1112–1115 (2018).

11. T. R. Vincent, M. Avramova, J. Canham, P. Higgins, N. Bilkey, S. T. Mugford, M. Pitino, M. Toyota, S. Gilroy, A. J. Miller, S. A. Hogenhout, D. Sanders, Interplay of plasma membrane and vacuolar ion channels, together with BAK1, elicits rapid cytosolic calcium elevations in *Arabidopsis* during aphid feeding. *Plant Cell* **29**, 1460–1479 (2017).

12. W. G. Choi, M. Toyota, S. H. Kim, R. Hilleary, S. Gilroy, Salt stress-induced $Ca^{2+}$ waves are associated with rapid, long-distance root-to-shoot signaling in plants. *Proc. Natl. Acad. Sci. U.S.A.* **111**, 6497–6502 (2014).

13. C. T. Nguyen, A. Kurenda, S. Stolz, A. Chételat, E. E. Farmer, Identification of cell populations necessary for leaf-to-leaf electrical signaling in a wounded plant. *Proc. Natl. Acad. Sci. U.S.A.* **115**, 10178–10183 (2018).

14. W. G. Choi, R. Hilleary, S. J. Swanson, S. H. Kim, S. Gilroy, Rapid, long-distance electrical and calcium signaling in plants. *Annu. Rev. Plant Biol.* **67**, 287–307 (2016).

15. J. Y. Lee, X. Wang, W. Cui, R. Sager, S. Modla, K. Czymmek, B. Zybaliov, K. van Wijk, C. Zhang, H. Lu, V. Lakshmanan, A plasmodesmata-localized protein mediates crosstalk between cell-to-cell communication and innate immunity in *Arabidopsis*. *Plant Cell* **23**, 3353 (2011), 3373.

16. C. L. Thomas, E. M. Bayer, C. Ritzenthaler, L. Fernandez-Calvino, A. J. Maule, Specific targeting of a plasmodesmal protein affecting cell-to-cell communication. *PLOS Biol.* **6**, e7 (2008).

17. A. Vaten, J. Dettmer, S. Wu, Y. D. Stierhof, S. Miyashima, S. R. Yadav, C. J. Roberts, A. Campilho, V. Bulone, R. Lichtenberger, S. Lehesranta, A. P. Mahonen, J. Y. Kim, E. Jokitalo, N. Sauer, B. Scheres, K. Nakajima, A. Carlsbecker, K. L. Gallagher, Y. Helariutta, Callose biosynthesis regulates symplastic trafficking during root development. *Dev. Cell* **21**, 1144–1155 (2011).

18. R. Tenorio Berrío, K. Verstaen, N. Vandamme, J. Pevernagie, I. Achon, J. Van Duyse, G. Van Isterdael, Y. Saeys, L. De Veylder, D. Inzé, M. Dubois, Single-cell transcriptomics sheds light on the identity and metabolism of developing leaf cells. *Plant Physiol.* **188**, 898–918 (2022).

19. A. Alfieri, F. G. Doccula, R. Pederzoli, M. Grenzi, M. C. Bonza, L. Luoni, A. Candeo, N. Romano Armada, A. Barbiroli, G. Valentini, T. R. Schneider, A. Bassi, M. Bolognesi, M. Nardini, A. Costa,

The structural bases for agonist diversity in an *Arabidopsis thaliana* glutamate receptor-like channel. *Proc. Natl. Acad. Sci. U.S.A.* **117**, 752–760 (2020).

20. N. Helassa, C. D. Dürst, C. Coates, S. Kerruth, U. Arif, C. Schulze, J. S. Wiegert, M. Geeves, T. G. Oertner, K. Török, Ultrafast glutamate sensors resolve high-frequency release at Schaffer collateral synapses. *Proc. Natl. Acad. Sci. U.S.A.* **115**, 5594–5599 (2018).

21. J. S. Marvin, B. G. Borghuis, L. Tian, J. Cichon, M. T. Harnett, J. Akerboom, A. Gordus, S. L. Renninger, T. W. Chen, C. I. Bargmann, M. B. Orger, E. R. Schreiter, J. B. Demb, W. B. Gan, S. A. Hires, L. L. Looger, An optimized fluorescent probe for visualizing glutamate neurotransmission. *Nat. Methods* **10**, 162–170 (2013).

22. E. M. Kramer, N. L. Frazer, T. I. Baskin, Measurement of diffusion within the cell wall in living roots of *Arabidopsis thaliana*. *J. Exp. Bot.* **58**, 3005–3015 (2007).

23. S. A. Mousavi, A. Chauvin, F. Pascaud, S. Kellenberger, E. E. Farmer, *GLUTAMATE RECEPTOR-LIKE* genes mediate leaf-to-leaf wound signalling. *Nature* **500**, 422–426 (2013).

24. L. Caarls, J. Elberse, M. Awwanah, N. R. Ludwig, M. de Vries, T. Zeilmaker, S. C. M. Van Wees, R. C. Schuurink, G. Van den Ackerveken, *Arabidopsis* jasmonate-induced oxygenases down-regulate plant immunity by hydroxylation and inactivation of the hormone jasmonic acid. *Proc. Natl. Acad. Sci. U.S.A.* **114,** 6388–6393 (2017)

25. E. Smirnova, V. Marquis, L. Poirier, Y. Aubert, J. Zumsteg, R. Ménard, L. Miesch, T. Heitz, Jasmonic acid oxidase 2 hydroxylates jasmonic acid and represses basal defense and resistance responses against botrytis cinerea infection. *Mol. Plant* **10**, 1159–1173 (2017).

26. Y. Liu, J. E. Ahn, S. Datta, R. A. Salzman, J. Moon, B. Huyghues-Despointes, B. Pittendrigh, L. L. Murdock, H. Koiwa, K. Zhu-Salzman, *Arabidopsis* vegetative storage protein is an anti-insect acid phosphatase, *Plant Physiol.* **139**, 1545–1556 (2005)

27. K. Pirc, L. A. Clifton, N. Yilmaz, A. Saltalamacchia, M. Mally, T. Snoj, N. Žnidaršič, M. Srnko, J. Borišek, P. Parkkila, I. Albert, M. Podobnik, K. Numata, T. Nürnberger, T. Viitala, J. Derganc, A.

Magistrato, J. H. Lakey, G. Anderluh, An oomycete NLP cytolysin forms transient small pores in lipid membranes. *Sci. Adv.* **8**, eabj9406 (2022).

28. D. A. Ammendolia, W. M. Bement, J. H. Brumell, Plasma membrane integrity: Implications for health and disease. *BMC Biol.* **19**, 71 (2021).

29. D. Tran, R. Galletti, E. D. Neumann, A. Dubois, R. Sharif-Naeini, A. Geitmann, J.-M. Frachisse, O. Hamant, G. C. Ingram, A mechanosensitive $Ca^{2+}$ channel activity is dependent on the developmental regulator DEK1. *Nat. Commun.* **8**, 1009 (2017).

30. S. E. Murthy, A. E. Dubin, T. Whitwam, S. Jojoa-Cruz, S. M. Cahalan, S. A. R. Mousavi, A. B. Ward, A. Patapoutian, OSCA/TMEM63 are an evolutionarily conserved family of mechanically activated ion channels. *eLife* **7**, e41844 (2018).

31. K. Yoshimura, K. Iida, H. Iida, MCAs in *Arabidopsis* are $Ca^{2+}$-permeable mechanosensitive channels inherently sensitive to membrane tension. *Nat. Commun.* **12**, 6074 (2021).

32. I. Radin, R. A. Richardson, J. H. Coomey, E. R. Weiner, C. S. Bascom, T. Li, M. Bezanilla, E. S. Haswell, Plant PIEZO homologs modulate vacuole morphology during tip growth. *Science* **373**, 586–590 (2021).

33. S. A. R. Mousavi, A. E. Dubin, W.-Z. Zeng, A. M. Coombs, K. Do, D. A. Ghadiri, W. T. Keenan, C. Ge, Y. Zhao, A. Patapoutian, PIEZO ion channel is required for root mechanotransduction in *Arabidopsis thaliana*. *Proc. Natl. Acad. Sci. U.S.A.* **118**, e2102188118 (2021).

34. S. Gilroy, M. Białasek, N. Suzuki, M. Górecka, A. R. Devireddy, S. Karpiński, R. Mittler, ROS, calcium, and electric signals: Key mediators of rapid systemic signaling in plants. *Plant Physiol.* **171**, 1606–1615 (2016).

35. J. L. Julien, J. M. Frachisse, Involvement of the proton pump and proton conductance change in the wave of depolarization induced by wounding in *Bidens pilosa*. *Can. J. Bot.* **70**, 1451–1458 (1992).

36. E. E. Farmer, Y. Q. Gao, G. Lenzoni, J. L. Wolfender, Q. Wu, Wound- and mechanostimulated electrical signals control hormone responses. *New Phytol.* **227**, 1037–1050 (2020).

37. J. M. Cheeseman, B. G. Pickard, Depolarization of cell membranes in leaves of *Lycopersicon* by extract containing Ricca's factor. *Can. J. Bot.* **55**, 511–519 (1977).

38. J. W. V. Sambeek, B. G. Pickard, C. E. Ulbright, Mediation of rapid electrical, metabolic, transpirational, and photosynthetic changes by factors released from wounds. II. Mediation of the variation potential by Ricca's factor. *Can. J. Bot.* **54**, 2651–2661 (1976).

39. S. J. Clough, A. F. Bent, Floral dip: A simplified method for *Agrobacterium*-mediated transformation of *Arabidopsis thaliana*. *Plant J.* **16**, 735–743 (1998).

40. J. Schindelin, I. Arganda-Carreras, E. Frise, V. Kaynig, M. Longair, T. Pietzsch, S. Preibisch, C. Rueden, S. Saalfeld, B. Schmid, J. Y. Tinevez, D. J. White, V. Hartenstein, K. Eliceiri, P. Tomancak, A. Cardona, Fiji: An open-source platform for biological-image analysis. *Nat. Methods* **9**, 676–682 (2012).

41. R Core Team (2022). R: A language and environment for statistical computing. R Foundation for Statistical Computing, Vienna, Austria. https://www.R-project.org/.

42. A. Zeileis, G. Grothendieck, Zoo: S3 infrastructure for regular and irregular time series. *J. Stat. Softw*. **14**, 1–27 (2005).

43. C. Faulkner, E. Petutschnig, Y. Benitez-Alfonso, M. Beck, S. Robatzek, V. Lipka, A. J. Maule, LYM2-dependent chitin perception limits molecular flux via plasmodesmata. *Proc. Natl. Acad. Sci. U.S.A.* **110**, 9166–9170 (2013).

44. J. R. Kikkert, The Biolistic PDS-1000/He device. *Plant Cell Tiss. Org. Cult.* **33**, 221–226 (1993).

45. M. G. Johnston, C. Faulkner, A bootstrap approach is a superior statistical method for the comparison of non-normal data with differing variances. *New Phytol.* **230**, 23–26 (2021).

46. J. Hellemans, G. Mortier, A. De Paepe, F. Speleman, J. Vandesompele, qBase relative quantification framework and software for management and automated analysis of real-time quantitative PCR data. *Genome Biol.* **8,** R19 (2007).

47. D. Bates, M.Maechler, B. Bolker, S. Walker, Fitting linear mixed-effects models using lme4. *J. Stat. Softw.*, **67**, 1–48 (2015).

48. R. V. Lenth, "emmeans: Estimated Marginal Means, aka Least-Squares Means," R package version 1.7.3 (2022); https://CRAN.R-project.org/package=emmeans.

49. S. Mielke, M. Zimmer, M. K. Meena, R. Dreos, H. Stellmach, B. Hause, C. Voiniciuc, D. Gasperini, Jasmonate biosynthesis arising from altered cell walls is prompted by turgor-driven mechanical compression. *Sci. Adv.* **7**, eabf0356 (2021).

50. A. Schulze, M. Zimmer, S. Mielke, H. Stellmach, C. W. Melnyk, B. Hause, D. Gasperini, Wound-induced shoot-to-root relocation of JA-Ile precursors coordinates *Arabidopsis* growth, *Mol. Plant*, **12**, 1383–1394 (2019).
